# Dual benefits of *CmNOR/Cmnor* heterozygous plants: prolonging shelf life and preserving fruit quality in oriental melon

**DOI:** 10.1093/hr/uhaf254

**Published:** 2025-09-22

**Authors:** Jinfang Wang, Ying Li, Shouwei Tian, Haiying Zhang, Yongtao Yu, Jie Zhang, Maoying Li, Yi Ren, Shengjin Liao, Chen Zhang, Guoyi Gong, Qing Wang, Yong Xu

**Affiliations:** State Key Laboratory of Vegetable Biobreeding, National Engineering Research Center for Vegetables, Beijing Key Laboratory of Crop Molecular Design and Intelligent Breeding Key Laboratory of Biology and Genetics Improvement of Horticultural Crops (North China), Beijing Vegetable Research Center, Beijing Academy of Agriculture and Forestry Science, Beijing 100097, China; Key Laboratory of Vegetable Postharvest Processing, Ministry of Agriculture and Rural Affairs, Beijing Key Laboratory of Fruits and Vegetable Storage and Processing, Key Laboratory of Biology and Genetic Improvement of Horticultural Crops (North China) of Ministry of Agriculture, Key Laboratory of Urban Agriculture (North) of Ministry of Agriculture, Institute of Agri-food Processing and Nutrition, Beijing Academy of Agriculture and Forestry Sciences, Beijing 100097, China; State Key Laboratory of Vegetable Biobreeding, National Engineering Research Center for Vegetables, Beijing Key Laboratory of Crop Molecular Design and Intelligent Breeding Key Laboratory of Biology and Genetics Improvement of Horticultural Crops (North China), Beijing Vegetable Research Center, Beijing Academy of Agriculture and Forestry Science, Beijing 100097, China; State Key Laboratory of Vegetable Biobreeding, National Engineering Research Center for Vegetables, Beijing Key Laboratory of Crop Molecular Design and Intelligent Breeding Key Laboratory of Biology and Genetics Improvement of Horticultural Crops (North China), Beijing Vegetable Research Center, Beijing Academy of Agriculture and Forestry Science, Beijing 100097, China; State Key Laboratory of Vegetable Biobreeding, National Engineering Research Center for Vegetables, Beijing Key Laboratory of Crop Molecular Design and Intelligent Breeding Key Laboratory of Biology and Genetics Improvement of Horticultural Crops (North China), Beijing Vegetable Research Center, Beijing Academy of Agriculture and Forestry Science, Beijing 100097, China; State Key Laboratory of Vegetable Biobreeding, National Engineering Research Center for Vegetables, Beijing Key Laboratory of Crop Molecular Design and Intelligent Breeding Key Laboratory of Biology and Genetics Improvement of Horticultural Crops (North China), Beijing Vegetable Research Center, Beijing Academy of Agriculture and Forestry Science, Beijing 100097, China; State Key Laboratory of Vegetable Biobreeding, National Engineering Research Center for Vegetables, Beijing Key Laboratory of Crop Molecular Design and Intelligent Breeding Key Laboratory of Biology and Genetics Improvement of Horticultural Crops (North China), Beijing Vegetable Research Center, Beijing Academy of Agriculture and Forestry Science, Beijing 100097, China; State Key Laboratory of Vegetable Biobreeding, National Engineering Research Center for Vegetables, Beijing Key Laboratory of Crop Molecular Design and Intelligent Breeding Key Laboratory of Biology and Genetics Improvement of Horticultural Crops (North China), Beijing Vegetable Research Center, Beijing Academy of Agriculture and Forestry Science, Beijing 100097, China; State Key Laboratory of Vegetable Biobreeding, National Engineering Research Center for Vegetables, Beijing Key Laboratory of Crop Molecular Design and Intelligent Breeding Key Laboratory of Biology and Genetics Improvement of Horticultural Crops (North China), Beijing Vegetable Research Center, Beijing Academy of Agriculture and Forestry Science, Beijing 100097, China; State Key Laboratory of Vegetable Biobreeding, National Engineering Research Center for Vegetables, Beijing Key Laboratory of Crop Molecular Design and Intelligent Breeding Key Laboratory of Biology and Genetics Improvement of Horticultural Crops (North China), Beijing Vegetable Research Center, Beijing Academy of Agriculture and Forestry Science, Beijing 100097, China; State Key Laboratory of Vegetable Biobreeding, National Engineering Research Center for Vegetables, Beijing Key Laboratory of Crop Molecular Design and Intelligent Breeding Key Laboratory of Biology and Genetics Improvement of Horticultural Crops (North China), Beijing Vegetable Research Center, Beijing Academy of Agriculture and Forestry Science, Beijing 100097, China; Key Laboratory of Vegetable Postharvest Processing, Ministry of Agriculture and Rural Affairs, Beijing Key Laboratory of Fruits and Vegetable Storage and Processing, Key Laboratory of Biology and Genetic Improvement of Horticultural Crops (North China) of Ministry of Agriculture, Key Laboratory of Urban Agriculture (North) of Ministry of Agriculture, Institute of Agri-food Processing and Nutrition, Beijing Academy of Agriculture and Forestry Sciences, Beijing 100097, China; State Key Laboratory of Vegetable Biobreeding, National Engineering Research Center for Vegetables, Beijing Key Laboratory of Crop Molecular Design and Intelligent Breeding Key Laboratory of Biology and Genetics Improvement of Horticultural Crops (North China), Beijing Vegetable Research Center, Beijing Academy of Agriculture and Forestry Science, Beijing 100097, China

## Abstract

Oriental melon, a climacteric fruit prized for its superior quality, faces limited shelf life. Although knockout *NON-RIPENING* (*CmNOR*) prolongs storage duration at the expense of quality loss, the potential of its direct agricultural application to reconcile this conflict remains uninvestigated. Through crossing homozygotes *Cmnor* and wild-type (WT) plants, we created *CmNOR/Cmnor* heterozygotes. These heterozygotes exhibited a 6-day ripening delay accompanied by reduced sucrose and β-carotene levels, yet ultimately attained WT quality parameters. Exogenous ethylene treatment accelerated fruit softening but failed to restore key quality parameters in both heterozygotes and homozygotes to WT levels. Transcriptomic and quantitative polymerase chain reaction (qPCR) analysis revealed that homozygotes displayed >10-fold expression differences versus WT in quality-associated genes (e.g. involved in carotenoid biosynthesis and sucrose metabolism). These expression disparities diminished to approximately 2-fold in heterozygotes. Furthermore, heterozygotes extended shelf life by 3–5 days during storage at 20°C while maintaining fruit quality. Storage-phase differential genes clustered in water regulation and cell wall modification pathways, with heterozygous-WT expression disparities gradually decreasing over time. The *CmNOR* dosage effect dynamically modulates interconnected quality and preservation networks, proposing an editing-based solution to overcome the storability-quality dichotomy in climacteric fruits.

## Introduction

Melon (*Cucumis melo* L.) is one of the important horticultural crops in the Cucurbitaceae family, and its cultivation area is increasing year by year. There are two main types of melon fruit: thick-rind cantaloupe (*C. melo* var. *cantaloupensis*) and thin-rind oriental melon (*C. melo* var. *makuwa* Makino), both of which are widely cultivated globally [1]. The oriental melon with thin-rind has gained considerable popularity due to the edibility of both its rind and flesh, offering a crispy and juicy texture [2]. This rise in consumer interest has led to the rapid development of its cultivation and its widespread adoption. However, thin-rind melon cultivars face more stringent geographical and climatic limitations than thick-rind cultivars. Additionally, the thin rind makes it prone to cracking, mechanical damage, and rot, which limits its suitability for long-term storage and transport. Consequently, thin-rind melons are primarily sold locally and have a short market shelf life. When harvested prematurely for the market, these melons tend to not be fully ripe, with lower sugar content, which affects their quality and marketability. Therefore, extending the shelf life of thin-rind melon while maintaining high fruit quality during production and postharvest storage is critical for increasing its economic value.

To address this issue, climacteric melon cultivars have been crossed with nonclimacteric cultivar via traditional genetic breeding methods, resulting in these climacteric melons with longer shelf life, but this typically comes at the cost of lower fruit quality and the need for a prolonged breeding cycle to stabilize the relevant desired traits [3–5]. Gene editing provides a promising solution by allowing precise modifications to specific genes, creating mutants with either complete or partial loss of gene function to realize the rapid acquisition of a target trait [6–9]. This approach has already been applied in some plant species with climacteric fruits and other crops that are prone to poor storage and transport. For instance, tomato (*Solanum lycopersicum*), which is a classic climacteric fruit, has been one focus of such applications. The natural spontaneous mutants *nonripening* (*nor*) is unable to accumulate lycopene as in the wild-type (WT) and maintains higher fruit hardness, resulting in exhibiting a significantly extended shelf life [6]. However, the fruits of *nac-nor* mutants edited in *NOR* can ripen and accumulate lycopene, but it does not reach the levels observed in the WT [6, 10]. Subsequently, gene-edited *nor* mutants with significantly extended shelf life have been reported in various horticultural crops. Knockout of *RIPENING INDUCING FACTOR (FvRIF)* in diploid strawberry (*Fragaria vesca*) or *ClNOR* in watermelon (*Citrullus lanatus*) significantly delays fruit ripening but reduces fruit quality [11, 12]. Knocking out *CmNAC-NOR* in climacteric thick-rind *Védrantais* melon plants also produces a similar delayed fruit ripening phenotype [8]. Similarly, knocking out *CmNOR* in climacteric thin-rind BYJH melon plants blocks fruit ripening and results in fruits with white flesh, lower sugar accumulation, and greater firmness, together with a prolonged shelf life [9]. Although these homozygous *Cmnor* mutants all exhibit an extended shelf life, their degrees of *NOR* mutant delay fruit ripening and extend shelf life to different levels, together with loss of fruit quality. This limitation restricts the direct application of knocking out *NOR* in melon production. Whether the dosage effect of the *NOR* gene in hybrid progeny can be utilized to create new varieties of thin-rind melons that balance shelf life and excellent quality of thin-rind melons has not yet been reported.

To address this industry challenge, we used the homozygous *Cmnor* mutants and crossed them with different high-quality parental lines with a functional *CmNOR*. The resulting heterozygous *CmNOR/Cmnor* hybrid plants demonstrated both an extended shelf life of over 6 days relative to the WT parents while maintaining fruit quality. Utilizing the homozygous *Cmnor* mutant as a parent therefore has the potential to extend the shelf life of thin-rind cantaloupes. Future research should focus on elucidating the specific increase in the number of days provided by the heterozygous *CmNOR/Cmnor* plants compared with mutants compared to the WT under both regular growth conditions and storage conditions.

## Results

### Heterozygous *CmNOR/Cmnor* fruits delayed fruit ripening and reached WT-like quality

In our previous research, we employed the clustered regularly interspaced short palindromic repeats (CRISPR)/CRISPR-associated nuclease 9 (Cas9) system to create *Cmnor* mutants [9]. The shelf life of fruits from homozygous *Cmnor* mutant plants was significantly longer than that of fruits from WT plants; however, fruit quality was notably lower, with fruits presenting paler and firmer flesh and lower sweetness. To address this quality loss, we crossed the *Cmnor* mutant line (harboring a 75-bp deletion in the NAC domain) with its parental line (BY9H) to produce heterozygous *CmNOR/Cmnor* F1 hybrids (Fig. S1A and B). We conducted a comparative analysis among WT, *Cmnor,* and *CmNOR/Cmnor* fruits during fruit ripening every 3 days from 30 to 45 days after pollination (DAP).

**Figure 1:**
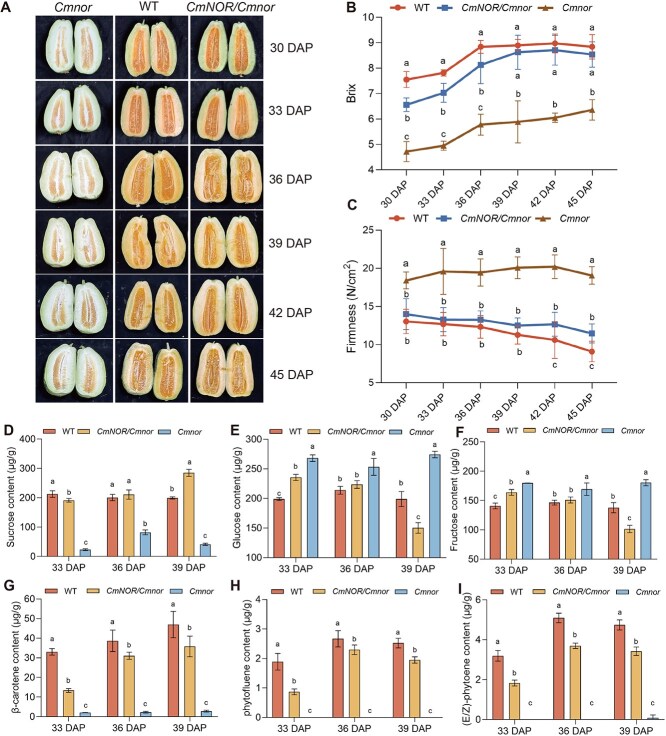
Phenotypic characterization of fruits from WT, *CmNOR/Cmnor*, and *Cmnor* plants during ripening. (A) Representative photographs of fruits from WT, *CmNOR/Cmnor*, and *Cmnor* fruits from 30 to 45 DAP. (B) Brix of WT, *CmNOR/Cmnor*, and *Cmnor* flesh from 30 to 45 DAP fruits. Three independent replicates were performed. (C) Firmness of WT, *CmNOR/Cmnor*, and *Cmnor* flesh from 30 to 45 DAP. Three independent replicates were performed. (D–F) Sucrose (D), glucose (E), and fructose (F) contents in the flesh of WT, *CmNOR/Cmnor*, and *Cmnor* fruits from 33 to 39 DAP. (G–I) β-Carotene (G), phytofluene (H), and (E/Z)-phytoene (I) contents in the flesh of WT, *CmNOR/Cmnor*, and *Cmnor* fruits from 33 to 39 DAP. In (B–I), values are means ± SD from three replicates. Asterisks denote significant differences compared with WT fruits at each ripening stage. Different lowercase letters indicate significant differences according to one-way ANOVA followed by Tukey’s multiple range test (*P* < 0.05).

At 30 DAP, the flesh of *CmNOR/Cmnor* and WT fruits was light green. The flesh of WT fruits turned completely orange by 33 DAP, while the flesh of *CmNOR/Cmnor* fruits was unevenly colored with light green and orange sectors, thus 6 days later than that of WT fruits. By 39 DAP, the *CmNOR/Cmnor* flesh turned completely orange; this flesh phenotype was similar to the WT flesh at 33 DAP. At 39 DAP, there was no clear difference in the flesh appearance of WT and *CmNOR/Cmnor* fruits. The flesh of *Cmnor* fruits remained pale during the entire time course (Fig. 1A). The °Brix of flesh from WT and *CmNOR/Cmnor* fruits increased from 30 to 36 DAP and stayed high at 8–9°Brix from 39 to 45 DAP, while *Cmnor* flesh was kept at 5–6°Brix from 30 to 45 DAP. The °Brix of flesh from *Cmnor* fruits was about 50% lower than that of WT fruits from 30 to 36 DAP, while that of *CmNOR/Cmnor* fruits was at most 15% lower over the same time period, with no significant differences detected between WT and *CmNOR/Cmnor* fruits from 39 DAP onward (Fig. 1B). During fruit ripening, the firmness of *CmNOR/Cmnor* and WT fruits gradually declined, while that of *Cmnor* fruits remained high, indicative of fruits with hard flesh. At 42 to 45 DAP, the firmness of flesh for *CmNOR/Cmnor* fruits was significantly higher than that of WT fruits (Fig. 1C). Compared with WT fruits, the development of *CmNOR/Cmnor* fruits was delayed by about 6 days, reaching WT levels after 39 DAP.

**Figure 2:**
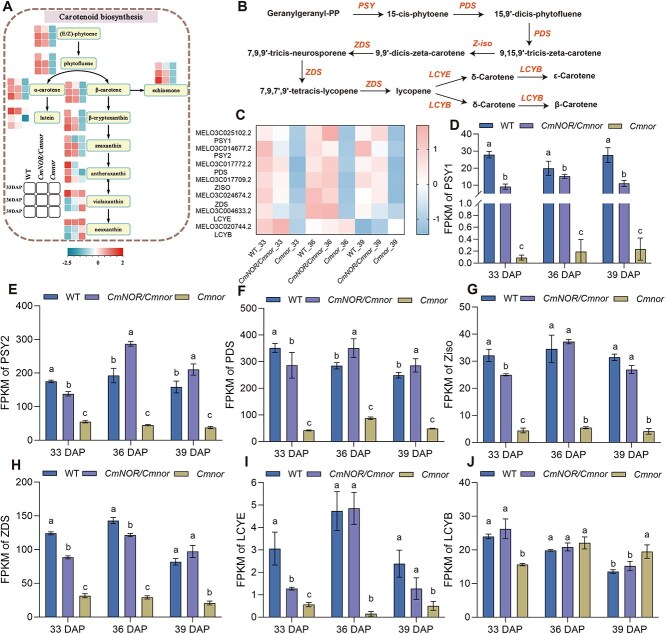
Analysis of the DEGs in the carotenoid biosynthesis pathway in WT, *CmNOR/Cmnor*, and *Cmnor* flesh from 33 to 39 DAP. (A) Diagram of the carotenoid biosynthesis pathway and relative abundance of differentially abundant metabolites in the fruits of WT, *CmNOR/Cmnor*, and *Cmnor* plants at 33, 36, and 39 DAP. In the heatmap, a value greater than zero indicates higher abundance; a value lower than zero indicates lower abundance. (B) Diagram of the carotenoid biosynthesis pathway showing the key genes and their associated metabolites. (C) Heatmap representation of expression levels for carotenoid biosynthesis genes in WT, *CmNOR/Cmnor*, and *Cmnor* flesh at 33, 36, and 39 DAP. In the heatmap, a value greater than zero indicates higher expression; a value lower than zero indicates lower expression. (D–J) Expression of key carotenoid biosynthesis genes in the flesh of WT, *CmNOR/Cmnor*, and *Cmnor* fruits at 33, 36, and 39 DAP. *PSY*, phytoene synthase (D, E); *PDS*, phytoene desaturase (F); *ZiSO*, 15-*cis*-zeta-carotene isomerase gene (G); *ZDS*, zeta-carotene desaturase (H); *LCYE*, lycopene ε-cyclase (I); *LCYB*, lycopene β-cyclase (J). Values are means ± SD from three independent replicates. Asterisks denote significant differences compared with WT fruits at each ripening stage. Different lowercase letters indicate significant differences according to Tukey’s multiple range test (*P* < 0.05).

Additionally, we quantified sugar and carotenoid levels in the three genotypes using LC–MS in the flesh of fruits from 33 to 39 DAP. For sugar content, a critical economic trait in melon fruits, we measured the levels of the primary sugar, sucrose, glucose, and fructose, with sucrose being the dominant contributor to sweetness. In *CmNOR/Cmnor* flesh, sucrose levels were 15% lower than those in WT flesh at 33 DAP, but this difference disappeared by 36 DAP (Fig. 1D). Strikingly, the flesh of *Cmnor* fruits exhibited persistently low sucrose levels, only accumulating about 10% of the levels seen in the flesh of *CmNOR/Cmnor* and WT fruits at 33 and 39 DAP. These results suggest that *CmNOR/Cmnor* undergoes delayed fruit ripening while maintaining flesh quality. Glucose and fructose levels exhibited similar trends as for sucrose. The levels of both sugars were elevated in the flesh of *CmNOR/Cmnor* and *Cmnor* fruits relative to WT fruits at 33 DAP, although they reached similar levels in the flesh of WT and *CmNOR/Cmnor* fruits at 36 DAP, with the flesh of *Cmno*r fruits still accumulating more of these sugars. At 39 DAP, glucose and fructose levels in the flesh of *CmNOR/Cmnor* fruits were about 50% those of WT fruits, while those in *Cmnor* flesh were higher than in WT fruits (Fig. 1E and F).

Consistent with the visual phenotypes of fruit flesh coloration, β-carotene levels, a key determinant of flesh color, were about 60% lower in *CmNOR/Cmnor* fruits compared with WT fruits at 33 DAP. Strikingly, *Cmnor* flesh contained barely detectable β-carotene levels (<20-fold lower relative to WT fruits) throughout the 33–39 DAP period (Fig. 1G). Three additional carotene metabolites, namely α-carotene, (E/Z)-phytoene, and phytofluene, followed a temporal pattern similar to that of β-carotene (Fig. 1H and I). Above all, the difference between *CmNOR/Cmnor* and WT fruits was narrowing from 36 DAP, and the final fruits reached WT level from 39 DAP.

Based on these results, we crossed the *Cmnor* mutant (in the BY9H background) with BY6H, a high-sucrose melon cultivar renowned for its sweet flavor, to generate F1 hybrids (*Cmnor* × BY6H and control WT × BY6H) (Fig. S1C and D). Phenotypic analysis revealed no discernible morphological differences between the fruits of these hybrid plants throughout ripening (Fig. S2A). However, physiological profiling uncovered notable divergences. °Brix levels in fruits of *Cmnor* × BY6H exhibited 30% lower than those in fruits from the corresponding WT × BY6H F1 hybrids at 30 and 33 DAP, but reached a comparable level at 36 DAP (Fig. S2B). Moreover, fruits from the *Cmnor* × BY6H F1 hybrids were significantly firmer (30% higher) than fruits from WT × BY6H F1 hybrids at 45 DAP (Fig. S2C). These results demonstrate that one copy of the *Cmnor* mutation delays ripening progression by approximately 6 days in a WT × BY6H F1 hybrid background, with normal sugar accumulation from 36 DAP onward, while textural maturation remains slower.

### The flesh of *CmNOR/Cmnor* fruits undergoes minor transcriptome changes compared with that of *Cmnor* fruits

From the metabolome analysis, we determined that 9 of 11 metabolites in the carotenoid pathway accumulate to significantly lower levels in *CmNOR/Cmnor* flesh compared with WT flesh at 33 DAP, before gradually increasing from 36 to 39 DAP, reaching levels closer to those seen in WT flesh (Fig. 2A). To elucidate the *CmNOR*-mediated regulatory network governing fruit quality, we conducted transcriptome profiling across critical ripening stages for the flesh of WT, *CmNOR/Cmnor*, and *Cmnor* fruits. Applying stringent criteria (|log2 fold-change| > 1, *P* < 0.05), pairwise comparisons revealed 1393–1451 differentially expressed genes (DEGs) between *CmNOR/Cmnor* and WT fruits at 33–39 DAP, contrasting with the substantially higher number of DEGs (7533–8291 DEGs) in *Cmnor* relative to WT fruits during equivalent developmental windows (Fig. S3A–C). Based on Venn diagrams looking at the overlap among gene lists, we identified a core regulatory hub comprising 816–1050 overlapping DEGs across all comparisons (Fig. S3D–F). A Kyoto encyclopedia of genes and genomes (KEGG) pathway enrichment analysis detected an enrichment of DEGs in three quality-determining pathways: starch–sucrose interconversion, carotenoid biogenesis, and phytohormone signaling cascades (Fig. S3G–I).

The 7 DEGs were mainly clustered in the carotene biosynthesis pathway (Fig. 2B and C). Notably, phytoene synthase paralogs *PSY1* (MELO3C025102.2) and *PSY2* (MELO3C014677.2) were expressed at very low levels in the pale flesh of *Cmnor* fruits at 33–39 DAP, showing ~4-fold and ~100-fold reduction, respectively, relative to WT fruits. In *CmNOR/Cmnor* fruits, *PSY1* reached a higher expression level than in WT fruits at 36–39 DAP (30%–80% higher), whereas *PSY2* remained lower than in WT fruit (≤40% WT levels), although being expressed at higher levels than in *Cmnor* fruit flesh (Fig. 2D). This partial recovery of gene expression extended to other genes in the carotenoid biosynthesis pathway: upstream genes including PDS (encoding phytoene desaturase, MELO3C017772.2), ZISO (encoding 15-*cis*-ζ-carotene isomerase, MELO3C017709.2), ZDS (encoding ζ-carotene desaturase MELO3C024674.2), and LCYE (encoding lycopene ε-cyclase, MELO3C004633.2) regained WT-like expression in *CmNOR/Cmnor* by 36 DAP, contrasting with their persistent repression in *Cmnor* (Fig. 2F–I). The expression of LCYB (encoding lycopene β-cyclase, MELO3C020744.2) showed a time-specific regulation among the genotypes, with LCYB expressed at 60% of WT levels in *Cmnor* fruit flesh at 33 DAP, similar to WT levels at 36 DAP, and 130% of WT levels at 39 DAP. LCYB was expressed at comparable levels in the flesh of WT and *CmNOR/Cmnor* fruits (Fig. 2J). The real-time qPCR (RT-qPCR) was performed to confirm the expression patterns of key carotenoid biosynthetic genes, and the results were consistent with the RNA-seq data (Fig. S4)*.* These changes in transcript levels mirrored the kinetics of carotenoid accumulation, mechanistically explaining the delayed pigmentation of *CmNOR/Cmnor* fruit flesh and the defective coloration of *Cmnor* fruits.

Sugar metabolites mainly clustered into four categories, namely pentose and glucuronate interconversions, galactose metabolism, starch and sucrose metabolism, and fructose and mannose metabolism (Fig. S5A). We explored the coordination of sucrose metabolism gene expression among WT, *CmNOR/Cmnor*, and *Cmnor* flesh (Fig. S5B and C). The expression of sucrose synthase gene (*Susy*, MELO3C025101.2), with a 7-fold induction in *Cmnor* and a 5-fold elevation in *CmNOR/Cmnor* at 33 DAP relative to WT fruits; this difference became attenuated between *CmNOR/Cmnor* and WT fruits at 36 DAP, with the heterozygous fruits exhibiting a 2-fold induction relative to WT fruits (Fig. S5D). The expressions of invertase gene *CmINV2* (MELO3C009488.2) and the trehalose-6-phosphate synthase gene *TPS* (MELO3C017588.2) were significantly induced in *Cmnor* and *CmNOR/Cmnor* flesh at 33 DAP, and dropped to WT levels from 36 DAP onward in *CmNOR/Cmnor* flesh, but remained high in the flesh of *Cmnor* fruits (Fig. S5E and G). The expressions of hexokinase gene (*HK*, MELO3C015169.2), the sugar transporter gene *Sugars will eventually be transported 3* (*SWEET3*, MELO3C005869.2) and the vacuolar sugar transporter (*VST1*, MELO3C013366.2), were expressed at much lower levels in *Cmnor* than in WT fruits, with *CmNOR/Cmnor* fruits exhibiting levels largely comparable with those of WT fruits (Fig. S5H and I). RT-qPCR was performed to confirm the expression patterns of key sugar metabolism genes, and the results were consistent with the RNA-seq data (Fig. S6). These transcript patterns suggested a compensatory induction of sucrose cleavage enzymes (Susy, INV) following the repression of transporter genes, with *CmNOR/Cmnor* often behaving like the WT through gene dosage effects.

When we examined the temporal expression patterns of genes encoding cell wall remodeling enzymes as a heatmap, the expression of xyloglucan endotransglucosylase/hydrolase genes (*XTH*) and polygalacturonase genes (PG) peaked at 33 DAP in the flesh of *CmNOR/Cmnor* fruits and *Cmnor* fruits, respectively, while pectinesterase genes (PE) peaked at 39 DAP in WT fruits (Fig. S7A). A *XTH* (MELO3C017481.2) and three *PG*s (MELO3C015128.2, MELO3C027277.2, and MELO3C016494.2) exhibited high expression in fruit flesh. We analyzed these four genes via RNA-seq and RT-qPCR. The *XTH* transcript level was 5-fold (33 DAP) and 10-fold (39 DAP) higher in *Cmnor* flesh compared to WT and *CmNOR/Cmnor* fruits (Fig. S7B and F). The three *PG*s showed coordinated upregulation, with significantly higher expression in *CmNOR/Cmnor* versus WT fruits (Figs S7C–S7E, S7G–S7I). In contrast, *PG* expression remained low in *Cmnor* flesh. These transcript patterns were associated with the firmness of three genetic lines during fruit ripening.

### Shelf life of *CmNOR/Cmnor* fruits is extended by about 3–5 days with minimal fruit quality loss

**Figure 3:**
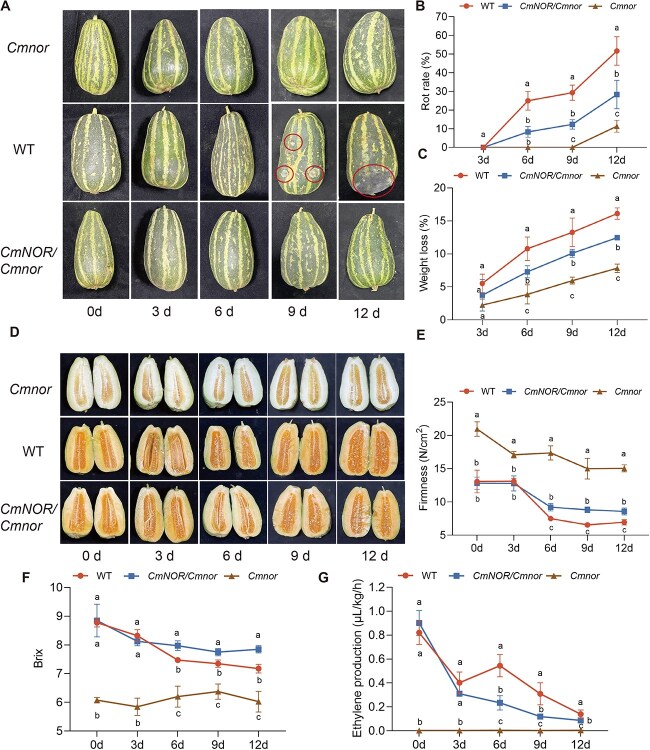
Postharvest phenotypic traits of WT, *CmNOR/Cmnor*, and *Cmnor* fruits at 36 DAP. **(**A) Representative photographs of fruits from WT, *CmNOR/Cmnor*, and *Cmnor* fruits from 0 to 12 days under storage conditions. The circles indicate rotting sectors. (B) Rot rate of WT, *CmNOR/Cmnor*, and *Cmnor* fruits over 12 days of storage. (C) Weight loss rate of the WT, *CmNOR/Cmnor*, and *Cmnor* fruits over 12 days of storage. (D) Representative photographs of cut fruits from WT, *CmNOR/Cmnor*, and *Cmnor* plants over 12 days of storage. (E) Firmness of WT, *CmNOR/Cmnor*, and *Cmnor* fruits over 12 days of storage. (F) Brix of WT, *CmNOR/Cmnor*, and *Cmnor* flesh over 12 days of storage. (G) Ethylene production of fruits from WT, *CmNOR/Cmnor*, and *Cmnor* plants over 12 days of storage. Values are means ± SD from nine replicates. Asterisks denote significant differences compared with WT fruits at each ripening stage, as determined by one-way ANOVA followed by Tukey’s multiple range test (*P* < 0.05).

To assess the shelf-life characteristics of the three genotypes, we subjected fruits harvested at full maturity (36 DAP) to postharvest storage at 20°C for 12 days. Distinct deterioration patterns emerged during monitoring. Visible quality decline initiated earlier in WT fruits, with pronounced surface wrinkling by Day 6 of storage (Fig. 3A). *CmNOR/Cmnor* fruits exhibited delayed deterioration, with initial symptoms manifesting on Day 9. We observed striking differences in decay progression: 100% of WT fruits and >50% of *CmNOR/Cmnor* fruits were completely rotten after 12 days of storage, with extensive mold development, whereas *Cmnor* fruits demonstrated significantly enhanced resistance to fungal infection throughout the storage period (Fig. 3A and B). As an indirect complementary measure of rind deterioration, we measured the amount of water lost in fruits over the 12 days of storage: WT fruits lost >15% of their initial mass during storage, in contrast to <10% for *CmNOR/Cmnor* fruits and <5% for *Cmnor* fruits. Notably, the differences in weight loss between WT and *CmNOR/Cmnor* fruits were statistically significant (*P* < 0.05) after at least 6 days of storage (Fig. 3C).

To quantify physiological changes postharvest, we monitored the soluble solid content (SSC) (°Brix), textural firmness, and ethylene evolution over time. While flesh color remained stable across genotypes throughout the storage period (Fig. 3D), °Brix and firmness values both gradually declined with time, indicating progressive quality deterioration (Fig. 3E and F). In WT fruits, the drop in firmness and °Brix was faster than that in *CmNOR/Cmnor* fruits after 6 days of storage onward, with 30% and 20% lower values, respectively, in WT fruits on Day 6 (Fig. 3E and F). The greater °Brix value suggests that *CmNOR/Cmnor* fruits maintain higher sugar reserves than WT fruits during storage. Notably, *CmNOR/Cmnor* fruits showed low ethylene production after 6 and 9 days of storage, whereas WT fruits displayed two peaks in ethylene production, one at the beginning of storage and another one on Day 6 (Fig. 3G). Quality degradation progressed with longer storage duration, with WT fruits demonstrating significantly faster deterioration rates than *CmNOR/Cmnor* and *Cmnor* fruits. The shelf life of *Cmnor* fruits exceeded that of *CmNOR/Cmnor* and WT fruits by >6 days, while *CmNOR/Cmnor* fruits maintained marketability for 3–5 days longer than WT fruits. This preservation pattern extended to the fruits of *Cmnor* × BY6H F1 hybrids, which showed a prolonged shelf life (3–5 days) compared with WT × BY6H F1 controls, concomitant with high °Brix values and a slower loss of firmness (Fig. S8).

### The expression levels of genes related to water responses are significantly higher in *CmNOR/Cmnor* flesh than in WT flesh during storage

**Figure 4:**
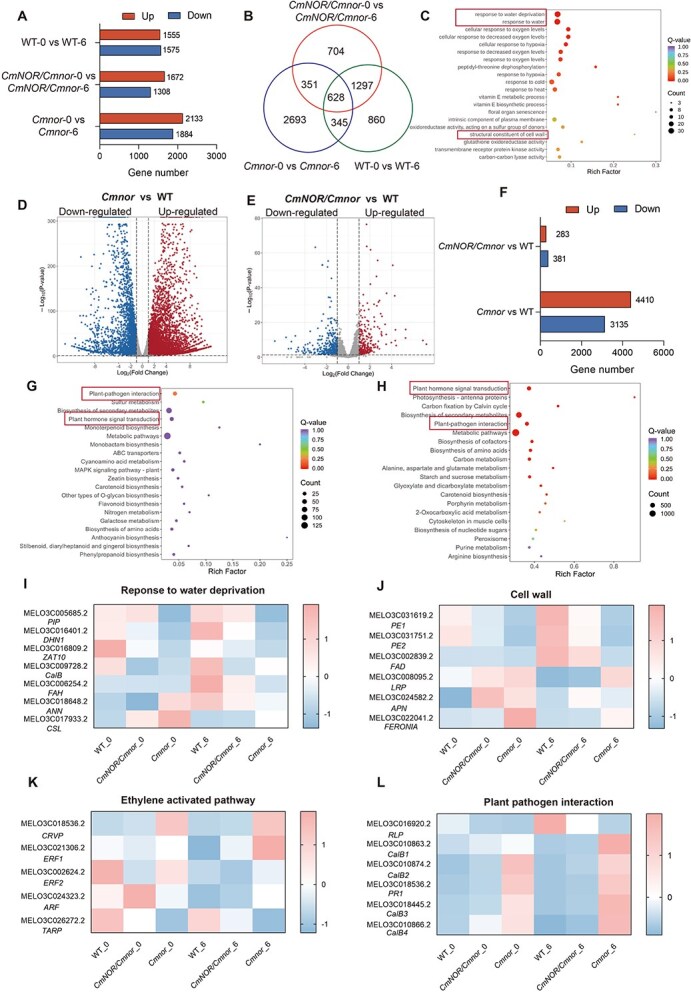
Analysis of DEGs in the flesh of WT, *CmNOR/Cmnor*, and *Cmnor* fruits during storage. (A) Number of downregulated or upregulated DEGs in the flesh of WT, *CmNOR/Cmnor*, and *Cmnor* fruits before storage versus after storage for 6 days. (B) Venn diagram showing the extent of overlap among the DEGs in the flesh of WT, *CmNOR/Cmnor*, and *Cmnor* fruits before storage versus after storage for 6 days. (C) Gene ontology (GO) term enrichment analysis of the overlapping genes in the flesh of WT, *CmNOR/Cmnor*, and *Cmnor* fruits. (D and E) Volcano plots showing the DEGs between WT and *Cmnor* (D) or WT and *CmNOR/Cmnor* (E) after 6 days of storage. (F) Number of downregulated or upregulated DEGs between WT, *CmNOR/Cmnor*, and *Cmnor* flesh. (G and H) KEGG pathway enrichment analysis of the DEGs between WT, *CmNOR/Cmnor*, and *Cmnor* fruit flesh. (I–L) Heatmap representation of expression levels for DEGs enriched in KEGG pathways related to water deprivation (I), cell wall biosynthesis (J), ethylene-activated pathway (K), and plant–pathogen interaction (L) in the flesh of WT, *CmNOR/Cmnor*, and *Cmnor* fruits before storage versus after storage for 6 days.

Our postharvest transcriptomic investigation revealed distinct preservation strategies among genotypes, with comparative analysis of WT, *CmNOR/Cmnor*, and *Cmnor* fruits before and after 6 days. There were 1555 upregulated/1575 downregulated (DEGs) in the group WT-0 versus WT-6, 1672 upregulated/1308 downregulated DEGs in the group *CmNOR/Cmnor*-0 versus *CmNOR/Cmnor*-6, and 2133 upregulated/1884 downregulated DEGs in the group *Cmnor*-0 versus *Cmnor*-6 (Fig. 4A). Six hundred twenty-eight shared DEGs among the pairwise comparisons of WT, *CmNOR/Cmnor*, and *Cmnor* fruits before and after a 6-day storage period (Fig. 4B and C). Striking differences emerged in storage-responsive transcriptomes: *Cmnor* fruit flesh exhibited massive transcriptome reprogramming (4410 upregulated and 3135 downregulated DEGs vs WT), dwarfing the limited perturbations in *CmNOR/Cmnor* fruit flesh (283 upregulated and 381 downregulated DEGs vs WT) (Fig. 4D–F). A KEGG pathway enrichment analysis converged on three enriched core mechanisms: plant–pathogen interactions, ethylene-activated signaling cascades, and carotenoid metabolism regulation (Fig. 4G and H).

We visualized the expression levels of postharvest regulatory genes as heatmaps, which revealed genotype-specific expression patterns associated with water retention capacity (Fig. 4I). Notably, the cellulose synthase gene *CSL* (MELO3C017933.2) exhibited about a 10-fold and 2-fold higher expression in *Cmnor* and *CmNOR/Cmnor* flesh, respectively, than in WT flesh after a 6-day storage period, suggesting enhanced cell wall reinforcement (Fig. S9A). Six stress-responsive genes displayed coordinated downregulation in *Cmnor* and *CmNOR/Cmnor*: the aquaporin gene *PIP* (MELO3C005685.2; 25-fold in *Cmnor*, 15% in *CmNOR/Cmnor*), the calmodulin-binding protein gene *CalB* (MELO3C009728.2; 2×/2×), the fatty acid hydroxylase gene *FAH* (MELO3C006254.2; 2000-fold/2-fold), the dehydrin gene *DHN1* (MELO3C016401.2; 20-fold/3-fold), the zinc finger protein gene *ZAT10* (MELO3C016809.2; 3-fold/1.5-fold), and the annexin gene ANN (MELO3C018648.2; 30% lower/20% lower) (Fig. S9B–G). RT-qPCR confirmed that the expression patterns of key genes involved in water deprivation response were consistent with the RNA-seq data (Fig. S9H–N).

We also observed genotype-specific postharvest adaptation strategies in the expression patterns of cell wall–associated pathways, as hinted by the GO analysis above. We identified six DEGs related to cell wall dynamics from a comparative analysis, divided into three repressed genes in *Cmnor* and *CmNOR/Cmnor* fruits, and three upregulated signaling-related genes in *Cmnor* and *CmNOR/Cmnor* fruits. The pectinesterase isoform genes *PE1* (MELO3C031619.2) and *PE2* (MELO3C031751.2), along with the oxidative metabolism regulator *FAD* (MELO3C002839.2), were barely expressed in *Cmnor* fruit flesh, while their transcript levels accumulated to about 50% of WT levels in *CmNOR/Cmnor* fruit flesh (Fig. S10A–C). Conversely, signaling components including the developmental regulator gene *LRP* (MELO3C008095.2), the protease gene *APN* (MELO3C024582.2), and the mechanosensitive kinase gene *FERONIA* (MELO3C022041.2) showed moderate 50% inductions in *CmNOR/Cmnor* after 6 days of storage (Fig. S10D–F). RT-qPCR confirmed that the expression patterns of key genes involved in cell wall organization were consistent with the RNA-seq data (Fig. S10G–L).

Other GO categories were related to ethylene biosynthesis and pathogen defense pathways, which again revealed genotype-specific postharvest adaptation mechanisms. Four ethylene-activated genes showed a progressive upregulation in *CmNOR/Cmnor* and *Cmnor*, while a tryptophan aminotransferase-related protein gene (*TARP*, MELO3C026272.2) was strongly repressed (10-fold in *Cmnor* and 2-fold in *CmNOR/Cmnor*) compared with expression in WT fruits (Fig. S11A). The expressions of key regulators such as ethylene-responsive transcription factor genes (*ERF1*, *ERF2*, MELO3C021306.2/MELO3C002624.2), an auxin-responsive factor gene (*ARF*, MELO3C024323.2), and a cysteine-rich venom protein gene (*CRVP*, MELO3C018536.2) showed dramatic induction in *Cmno*r (3- to 5-fold over WT levels), with *CmNOR/Cmnor* exhibiting intermediate expression levels (2-fold over WT levels) (Fig. S11B–E). RT-qPCR confirmed that the expression patterns of key genes in the ethylene signaling pathway were consistent with the RNA-seq data (Fig. S11F–J).

Concurrently, plant–pathogen interaction pathways revealed a striking genotype divergence. The expression levels of the pathogenesis-related protein gene PR1 (MELO3C018536.2) and four calcium sensor genes (ClaB1–4, MELO3C010863.2, MELO3C010874.2, MELO3C018445.2, MELO3C010866.2) were induced in *CmNOR/Cmnor* about 5- to 7-fold over those of WT fruits and 20-fold in *Cmnor* fruits. A receptor-like protein kinase gene (RLP, MELO3C016920.2) showed an opposite pattern, with a 40-fold suppression in *Cmnor* and a 4-fold suppression in *CmNOR/Cmnor* relative to WT fruits (Fig. S12A–F). RT-qPCR confirmed that the expression patterns of key genes involved in water deprivation response were consistent with the RNA-seq data (Fig. S12G–L).

These coordinated changes of enhanced ethylene responsiveness and reconfigured pathogen defenses collectively underpin *CmNOR/Cmnor* commercial superiority, synergistically extending shelf life through delayed ripening and reinforced biotic resistance while maintaining flesh integrity.

### Ethylene treatment accelerates fruit softening and shortens shelf life without compromising fruit quality

**Figure 5:**
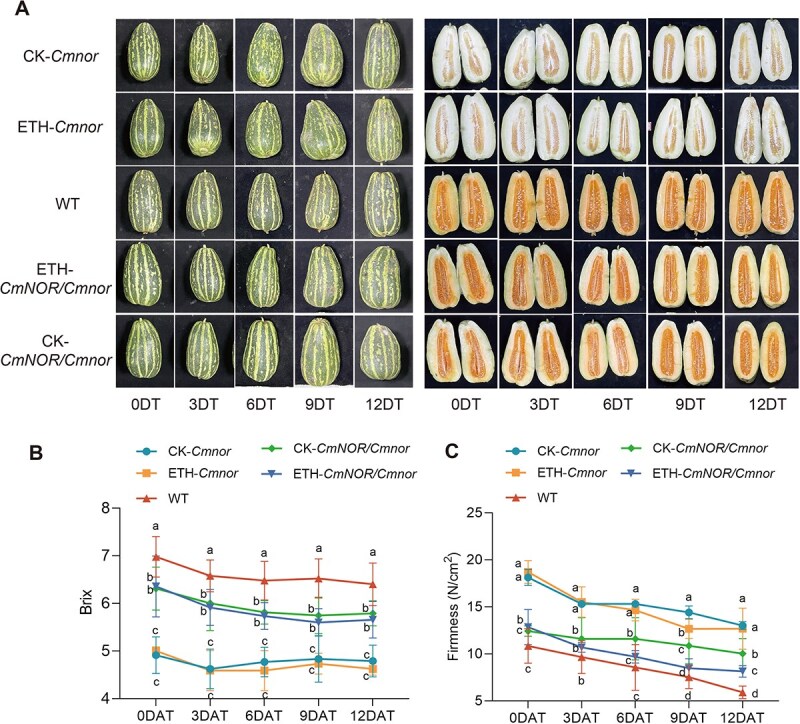
Ethylene treatment accelerates fruit softening. (A) Representative photographs of WT, *CmNOR/Cmnor*, and *Cmnor* fruits with or without ethylene treatment, over 12 days of storage (DT). (B) The °Brix of WT, *CmNOR/Cmnor*, and *Cmnor* fruits with or without ethylene treatment, over 12 days of storage. (C) The firmness of WT, *CmNOR/Cmnor*, and *Cmnor* fruits with or without ethylene treatment, over 12 days of storage. For (B, C), values are means ± SD from nine replicates. Asterisks denote significant differences compared with WT fruits at each ripening stage, as determined by one-way ANOVA followed by Tukey’s multiple range test (*P* < 0.05).

*CmNOR/Cmnor* and *Cmnor* fruits displayed significantly lower ethylene production at some time points (*CmNOR/Cmnor*) or throughout (*Cmnor*) the storage period. To investigate the regulatory role of ethylene in postharvest fruit quality, we treated 30 DAP *Cmnor* and *CmNOR/Cmnor* fruits with ethephon. Following treatment with ethylene or water as control and a 12-day storage period, we examined all fruits. Notably, ethylene treatment failed to induce pericarp darkening in either *CmNOR/Cmnor* or *Cmnor* fruits (Fig. 5A) and had no significant effect on the SSC (°Brix) compared with untreated controls (Fig. 5B). However, ethylene treatment accelerated the softening of *CmNOR/Cmnor* fruits. While *Cmnor* fruits maintained superior firmness relative to *CmNOR/Cmnor* and WT fruits regardless of treatment, the firmness of ethylene-treated *CmNOR/Cmnor* fruits declined to reach levels comparable with those of WT fruits not treated with ethylene (Fig. 5C). These findings collectively demonstrate that ethylene treatment accelerates textural deterioration without modifying coloration or biochemical quality parameters, ultimately leading to shortened shelf stability of *CmNOR/Cmnor* and *Cmnor* fruits.

## Discussion

Melons display a unique dual ripening physiology, exhibiting characteristics of both climacteric and nonclimacteric fruit types [4]. Thin-rind melon varieties predominantly follow a climacteric ripening pattern, characterized by a pronounced ethylene biosynthesis burst accompanied by rapid fruit softening, which substantially compromises postharvest longevity. In our previous investigation, we successfully generated homozygous *Cmnor* mutants in thin-rind melon through CRISPR/Cas9 editing. While these engineered lines demonstrated remarkably extended shelf-life exceeding that of conventional varieties, they also exhibited significant quality deterioration, particularly in their flavor profile and textural attributes [9]. To harness this genetic material for practical agricultural solutions, we generated heterozygous *CmNOR/Cmnor* plants through genetic crossing of the CRISPR/Cas9-derived homozygous *Cmnor* mutant to its WT parental line. Remarkably, the fruits of the resulting *CmNOR/Cmnor* F1 hybrids showed delayed ripening and a 3- to 5-day extension in their postharvest shelf life relative to WT fruits, while maintaining good fruit quality, including SSC and firmness (Figs S1 and S3). Furthermore, to optimize breeding outcomes, we crossed the *Cmnor* mutant with elite parental lines with superior organoleptic traits. The resulting F1 hybrids retained the extended shelf life without compromising key quality attributes, demonstrating the compatibility of the *Cmnor* mutant for generating high-yielding, consumer-preferred phenotypes (Figs S1 and S3). This synergistic approach of combining precision gene editing with strategic hybridization should substantially accelerate breeding cycles compared with conventional methods. These findings provide a mechanistic framework for developing new thin-skinned melon hybrid varieties with enhanced storability and transport resilience, addressing a critical bottleneck in commercial melon production.

Flesh pigmentation is a primary visual determinant of fruit quality, with coloration intensity directly influencing consumer appeal. This study focused on two thin-rind melon varieties characterized by orange mesocarp. Ripening-associated color transition progressed from white to orange, with fruit flesh of F1 hybrids exhibiting a 6-day delay in color shift compared with that of WT fruits, achieving comparable pigmentation by 39 DAP (Fig. 1). During postharvest storage, we did not detect any pronounced alterations in coloration or in the expression of carotenoid biosynthesis genes between *CmNOR/Cmnor* and WT fruits. We propose that the stabilization of flesh coloration prior to storage reflects the completion of β-carotene accumulation during ripening, entering a metabolic equilibrium phase where extended storage fails to stimulate further carotenogenesis.

Soluble sugar concentrations are pivotal to fruit quality, exhibiting dynamic changes throughout fruit developmental and postharvest phases. During fruit ontogeny, substantial sugar accumulation occurs, with pronounced divergence observed between F1 hybrids and their WT counterparts at early developmental stages (Fig. 1). This disparity progressively diminishes, with °Brix reaching similar levels in WT and *CmNOR/Cmnor* fruits by 39 DAP, reflecting the converging expression patterns of core sugar metabolism genes. Notably, *CmNOR/Cmnor* fruits demonstrated intermediate transcriptome profiles for these regulatory genes compared with *Cmnor* and WT fruits, potentially explaining their transitional sugar accumulation phenotypes (Fig. S3). Postharvest sugar dynamics diverge fundamentally from developmental accumulation patterns. In starch-accumulating species (e.g. apple, banana), transient sugar elevation occurs through the conversion of starch into soluble sugars during early storage, followed by progressive carbohydrate depletion with prolonged storage [13, 14]. By contrast, sucrose-dominated systems exemplified by thin-skinned melons exhibit rapid disaccharide degradation during storage, leading to marked quality deterioration. Our findings reveal that *CmNOR/Cmnor* fruits demonstrate attenuated sucrose degradation kinetics compared with WT fruits, effectively maintaining superior organoleptic properties. This preservation mechanism likely stems from hybrid-specific regulation of sucrose metabolism pathways, although the exact molecular mediators require elucidation.

**Figure 6:**
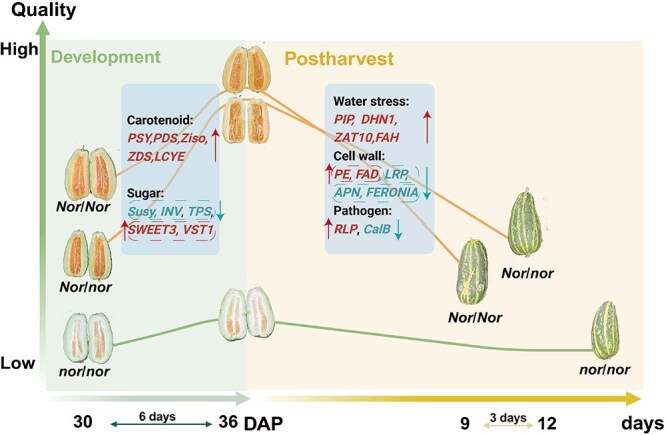
Proposed model of different *CmNOR* variants during fruit ripening and postharvest storage. *CmNOR/Cmnor* represents the WT lines, *CmNOR/Cmnor* represents heterozygous plants, and Cmnor/*Cmnor* are homozygous *Cmnor* mutants. The upward/downward arrow represents the *higher/lower* expression of genes in WT or *CmNOR/Cmnor* flesh than in *Cmnor* flesh, respectively.

Fruit firmness serves as a critical physiological marker for evaluating maturity and postharvest longevity, with established correlations between these two aspects across diverse species [15–17]. In melons, the *Cmnor* mutation confers a distinct firmness trajectory: while WT fruits undergo rapid softening during ripening, the firmness decline in *CmNOR/Cmnor* fruits is significantly slower, displaying intermediate firmness between homozygous *Cmno*r and WT fruits. This phenotypic gradient highlights the dosage effect of *Cmnor* on ripening regulation. Cell wall remodeling via enzymes such as polygalacturonases and pectin methylesterases drives tissue softening. Our comparative transcriptome analysis revealed that *CmNOR/Cmnor* fruits exhibit lower expression of these cell wall modification genes during fruit ripening and storage, mirroring findings in tomato, where *SlNOR* directly suppresses cell wall catabolism [10, 18]. The flesh of *CmNOR/Cmnor* fruits demonstrated intermediate repression of expression, delaying cell wall degradation and extending firmness retention. Slower water loss in *CmNOR/Cmnor* fruits (10% loss, in contrast to 15% for WT fruits) synergizes with delayed cell wall disassembly, collectively preserving structural integrity. This dual mechanism explains the extended shelf life of *CmNOR/Cmnor* fruits without compromising quality metrics, as observed in *Cmnor* lines.

The postharvest utilization of ethylene and 1-methylcyclopropene (1-MCP) has revolutionized the storage protocols for climacteric fruits by modulating their ripening trajectories. The application of these compounds to fruits profoundly alters their shelf life, with ethylene accelerating senescence while 1-MCP effectively delaying ripening [19, 20]. Ethylene treatment typically induces characteristic ripening phenotypes in climacteric species, including enhanced pigmentation, soluble sugar accumulation, and tissue softening through cell wall modifications [14]. This regulatory effect is exemplified in tomato *nor* mutants, where ethylene exposure partially restores ripening capacity by accelerating color transition and textural changes, ultimately shortening shelf stability [18]. By contrast, *Cmnor* homozygous mutants exhibit severely impaired ripening capacity, maintaining prolonged postharvest integrity but developing inferior organoleptic qualities [8, 9]. Intriguingly, while ethylene treatment significantly enhances mesocarp softening in these mutant melon fruits, it fails to rescue normal ripening parameters or restore characteristic sugar profiles and coloration development. Our experimental data corroborate this phenomenon across three distinct genotypes, demonstrating the consistent promotion of textural modification conferred by ethylene treatment without concomitant improvements in flesh coloration or carbohydrate metabolism (Fig. 3). These observations underscore the complex, species-specific nature of ethylene signaling, particularly regarding its dissociation between softening and other ripening markers. We speculate that this may be because the regulation of flesh color and sugar content in melon fruits relies on *CmNOR*, with key functional genes being directly or indirectly regulated by NOR. However, fruit firmness is additionally governed by other critical regulatory factors besides *CmNOR*, resulting in the differential response. Nevertheless, the precise molecular mechanisms governing ethylene-mediated fruit softening remain elusive, warranting further investigation into its downstream effectors and crosstalk with parallel ripening pathways.

The *CmNOR/Cmnor* F1 hybrids generated in this study demonstrated dual agronomic advantages, exhibiting a 6-day ripening delay and a 3- to 5-day shelf-life extension relative to WT fruits, while preserving key quality attributes (Fig. 6). This genotype-specific decoupling of ripening chronology from quality deterioration provides a strategic framework for breeding thin-skinned melon cultivars with enhanced postharvest integrity. Crucially, the delayed yet complete maturation in F1 hybrids ensured full flavor and pigment development prior to storage, marking a critical advancement over conventional ripening-inhibited mutants that often sacrifice sensory quality for longevity. These findings establish *CmNO*R heterozygosity as a promising target for molecular breeding programs aimed at optimizing the commercial viability of perishable cucurbit crops.

## Materials and methods

### Plant materials, treatments, and collection of samples

The oriental melon plants used in this study were in the cultivar ‘Boyangjiuhao’ (BY9H) and consisted of three types: homozygous *Cmnor* mutant generated by CRISPR in a previous study [9], heterozygous *CmNOR/Cmnor*, and ‘BY9H’ (WT). All three types were detected via the specific primers shown in Table S1. Melons were harvested at 30, 33, 36, 39, 42, and 45 DAP. Only melons that were uniform in size and free from pests and mechanical injuries were selected and arranged neatly in frames. For fruit ripening observation, three to five fruits per ripening stage were harvested for maturation analysis. They were then immediately transported to the Beijing Academy of Agricultural and Forestry Sciences for experimental work.

Fifteen 36 DAP melons of each type were chosen for sampling, and six were selected for the observation of storage phenotype. These melons were stored together in a cold room at 20°C with 85%–90% relative humidity. Samples were taken at 0, 3, 6, 9, and 12 days of storage, during which the main physiological indices, such as SSC and hardness, were measured. The rind and flesh of the melons were separated, and the flesh was cut into small, uniform pieces, frozen with liquid nitrogen, and stored at −80°C in an ultra-low temperature freezer for further analysis. Three melons of each genotype were sampled at each time point.

### Weight loss

Melon weight loss (%) was determined by weighing each fruit at each sampling time and calculating the percentage change relative to its initial weight. The same samples were used throughout the experiment to ensure consistency. Weight loss (WL) was calculated using the following formula: WL (%) = (IW − FW)/IW  × 100, where IW represents the initial weight and FW represents the final weight of the fruit [21].

### Determination of percentage of rotten fruits

Fruits exhibiting visible mold growth were classified as rotten fruits. Rotten fruits were counted every 3 days, and the percentage of rotten melons was calculated using the following formula: Rotten rate (%) = Number of rotten fruits/Total number of fruits × 100%.

### Sugar and carotenoid content measurements

SSC was quantified with a hand-held refractometer (Atago PAL-1, Tokyo, Japan) and expressed in °Brix (%), following the methodology of Wang *et al.* [22]. Sugar contents were determined by gas chromatography–mass spectrometry (GC–MS) on an Agilent 8890-5977B platform. Carotenoid contents were measured by liquid chromatography tandem mass spectrometry (LC–MS/MS) on an AB Sciex QTRAP 6500 platform by Metware Biotechnology (http://www.metware.cn/). Fruits were sampled from WT (BY9H), *CmNOR/Cmnor*, and *Cmnor* plants at 30, 33, 36, and 39 DAP. Three biological replicates were performed.

### Firmness measurement

Firmness was measured with a GY-3 hardness tester (Zhejiang Top Instrument Co., Ltd, China) at three equally spaced points at the equator of the melons. Measurements were expressed as mean values, following the procedure detailed by Jang *et al*. [23].

### Ethylene production

Ethylene release rates were determined following the method described by Guan *et al.* [24]. Ethylene production was measured using a gas chromatograph (Agilent Technologies 7820A, Santa Clara, CA, USA). Three melons for each genotype were sealed in a 9-l lockbox at 20°C for 1 h. Gas samples were collected, and the ethylene content was analyzed based on the linear relationship between peak area and concentration. The experiment was performed three times, with ethylene yield expressed as microliter per kilogram per hour.

### Transcriptome analysis

The method followed for the transcriptome analysis was described previously [9]. Briefly, RNA-seq libraries of flesh from WT, *Cmnor*, and *CmNOR/Cmnor* fruits were generated using a TruSeq RNA Sample Prep Kit (Illumina, USA) and sequenced on an Illumina sequencing platform by Metware Biotechnology. The clean reads were aligned to the Melon (DHL92) v3.6.1 reference genome via HISAT2. Gene expression levels were normalized as fragments per kilobase of transcript per million mapped reads (FPKM). DEGs were identified based on the criteria *P*-adjust <0.05 and |log2 fold-change| ≥ 1 via analysis on the free online Metware platform (https://cloud.metware.cn/).

### RT-qPCR analysis

Total RNA was extracted from melon flesh of different types with a kit (Huayueyang Biotech, Beijing, China). For RT-qPCR analysis, total RNA was reverse transcribed to first-strand cDNA with FastKing gDNA Dispelling RT SuperMix (Tiangen Biotech, Beijing, China). Quantitative PCR (qPCR) was performed following the previous study [12, 25]. The melon *CmCYP7* gene was used as the internal control followed by our previous studies [3, 9]. The specific primers were listed in Table S1.

### Exogenous ethylene treatment

To study the effect of ethylene treatment on melon quality, melons collected at 30 DAP from *Cmnor* and *CmNOR/Cmnor* plants were divided into two groups, each group comprising 15 melons for sampling and 6 for photography. For the group treated with ethylene followed by Hu *et al.* [26], the fruits were immersed in 1 g/l ethephon solution for 10 min, and then air-dried. For the control group, the fruits were immersed in distilled water for 10 min, and then air-dried. After treatment, melons were stored at 20°C in 0.03 PE film for 24 h before cutting them open lengthwise. Hardness, SSC, and photographs were recorded after 0, 3, 6, 9, and 12 days of storage.

## Supplementary Material

Web_Material_uhaf254
