# Supplementary material for: Dual benefits of CmNOR/Cmnor heterozygous plants: prolonging shelf life and preserving fruit quality in oriental melon
Source: Hortic Res. 2025 Sep 22;13(1):uhaf254. doi: 10.1093/hr/uhaf254 (PMC12858252; doi:10.1093/hr/uhaf254)
Supplement: Web_Material_uhaf254 [file web_material_uhaf254.docx]

**Dual Benefits of *CmNOR/Cmnor*** **Heterozygous Plants: Prolonging Shelf Life and Preserving Fruit Quality in Oriental Melon**

Jinfang Wang^1#^, Ying Li^2#^, Shouwei Tian^1^, Haiying Zhang^1^, Yongtao Yu^1^, Jie Zhang^1^, Maoying Li^1^, Yi Ren^1^, Shengjin Liao^1^, Chen Zhang^1^, Guoyi Gong^1^, Qing Wang^2,^* and Yong Xu^1,^*

1 State Key Laboratory of Vegetable Biobreeding, National Engineering Research Center for Vegetables, Beijing Key Laboratory of Crop Molecular Design and Intelligent Breeding Key Laboratory of Biology and Genetics Improvement of Horticultural Crops (North China), Beijing Vegetable Research Center, Beijing Academy of Agriculture and Forestry Science, Beijing, 100097, China

2 Key Laboratory of Vegetable Postharvest Processing, Ministry of Agriculture and Rural Affairs, Beijing Key Laboratory of Fruits and Vegetable Storage and Processing, Key Laboratory of Biology and Genetic Improvement of Horticultural Crops (North China) of Ministry of Agriculture, Key Laboratory of Urban Agriculture (North) of Ministry of Agriculture, Institute of Agri-food Processing and Nutrition, Beijing Academy of Agriculture and Forestry Sciences, Beijing, 100097, China

Corresponding author: [xuyong@nercv.org](mailto:xuyong@nercv.org) and wangqing@iapn.org.cn

# These authors contribute equally to this article.


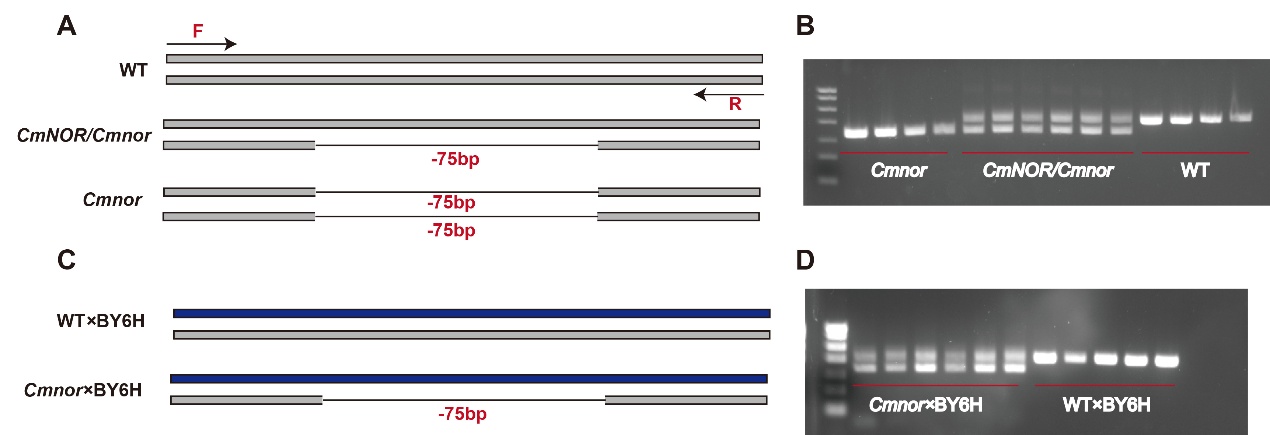


**Figure S1. Detection of the heterozygous and homozygous plants.**

A. Distribution of 75bp in WT, heterozygous and homozygous *Cmnor* plants.

B. PCR detection of three types.

C. Distribution of 75bp in WT×BY6H and *Cmnor*×BY6H plants.

D. PCR detection of WT×BY6H and *Cmnor*×BY6H plants.

**
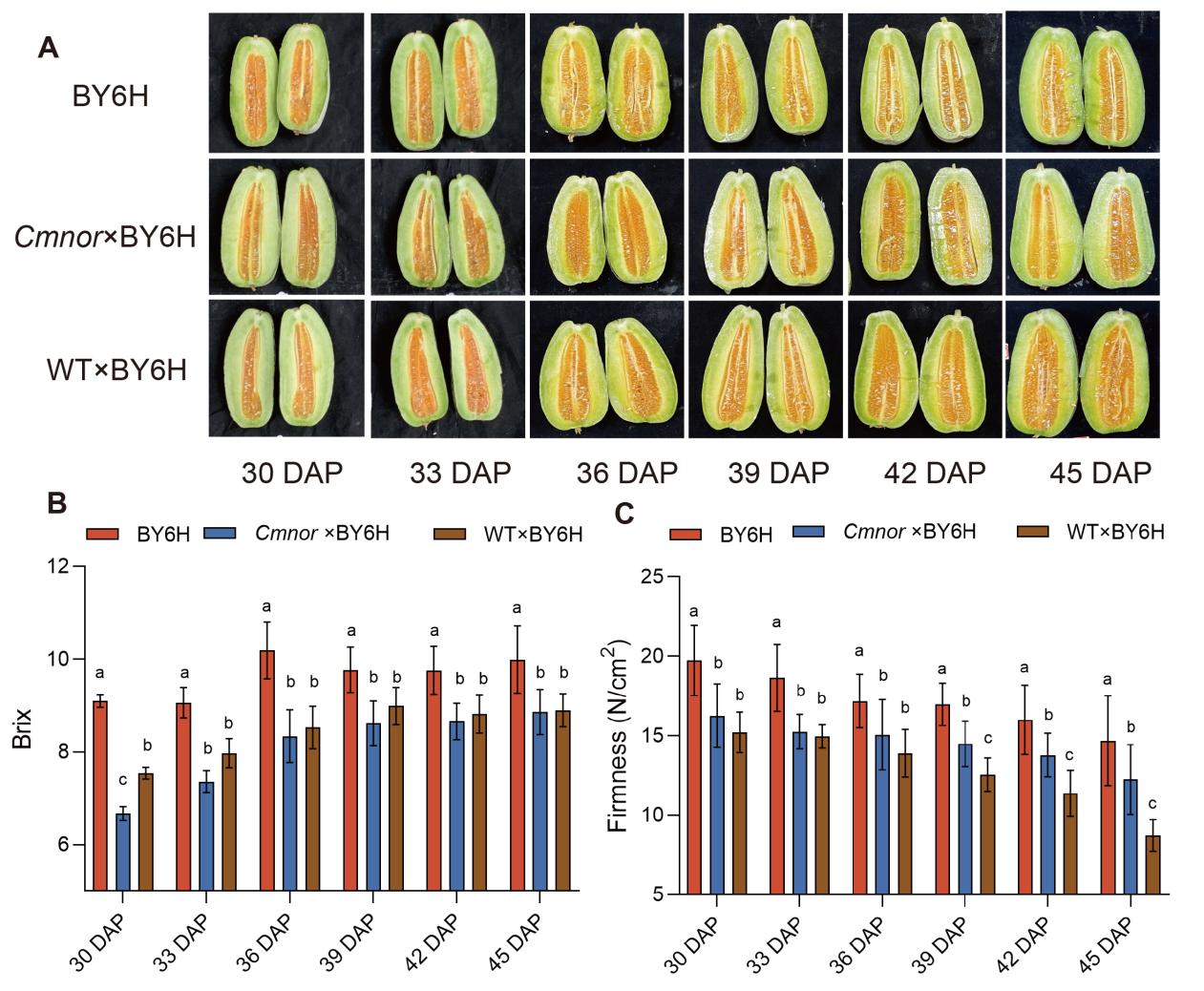
**

**Figure S2. Phenotypic characterization of fruits from BY6H, *Cmnor* (BY9H)×BY6H F1, andWT×BY6H F1 plants during fruit ripening.**

1. Representative photographs of fruits from BY6H, *Cmnor* (BY9H)×BY6H F1, and BY9H×BY6H F1 plants from 30 to 45 days after pollination (DAP).
2. °Brix of fruit flesh from BY6H, *Cmnor* (BY9H)×BY6H F1, and WT×BY6H F1 plants from 30 to 45 DAP. Values are means ± SD from three independent replicates. Different lowercase letters indicate significant differences according to Tukey’s multiple range test (*p* < 0.05).
3. Firmness of fruit flesh from BY6H, *Cmnor* (BY9H)×BY6H F1, and WT×BY6H F1 plants from 30 to 45 DAP. Values are means ± SD from three independent replicates. Asterisks denote significant differences compared with WT fruits at each ripening stage. Different lowercase letters indicate significant differences according to one-way ANOVA following by Tukey’s multiple range test (*p* < 0.05).

**
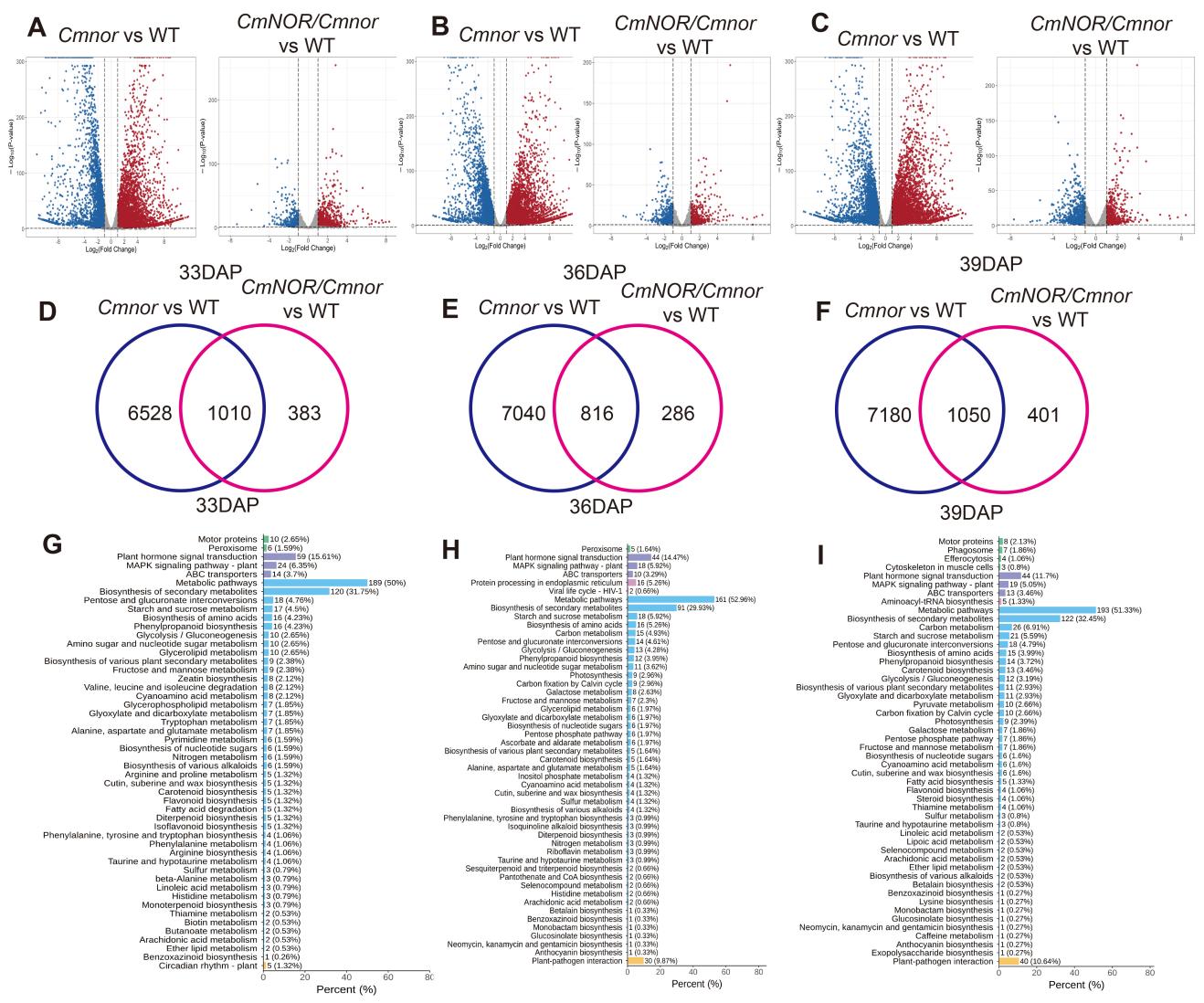
**

**Figure S3. Analysis of the differentially expressed genes between the flesh of WT, *CmNOR/Cmnor*, and *Cmnor* fruits at 33, 36, and 39 days after pollination.**

1. **C.** Volcano plots showing the differentially expressed genes (DEGs) in the flesh of WT, *CmNOR/Cmnor*, and *Cmnor* fruits at 33 (A), 36 (B), or 39 days after pollination (DAP) (C). Red circles represent upregulated genes; blue circles represent downregulated genes.
2. **F**. Venn diagrams showing the extent of overlap between the DEGs in the flesh of WT, *CmNOR/Cmnor*, and *Cmnor* fruits at 33 (D), 36 (E), or 39 DAP (F).
3. **I.** KEGG pathway enrichment analysis of the overlapping DEGs between *CmNOR/Cmnor* vs. WT and *Cmnor* vs. WT at 33 (G), 36 (H), or 39 DAP (I).


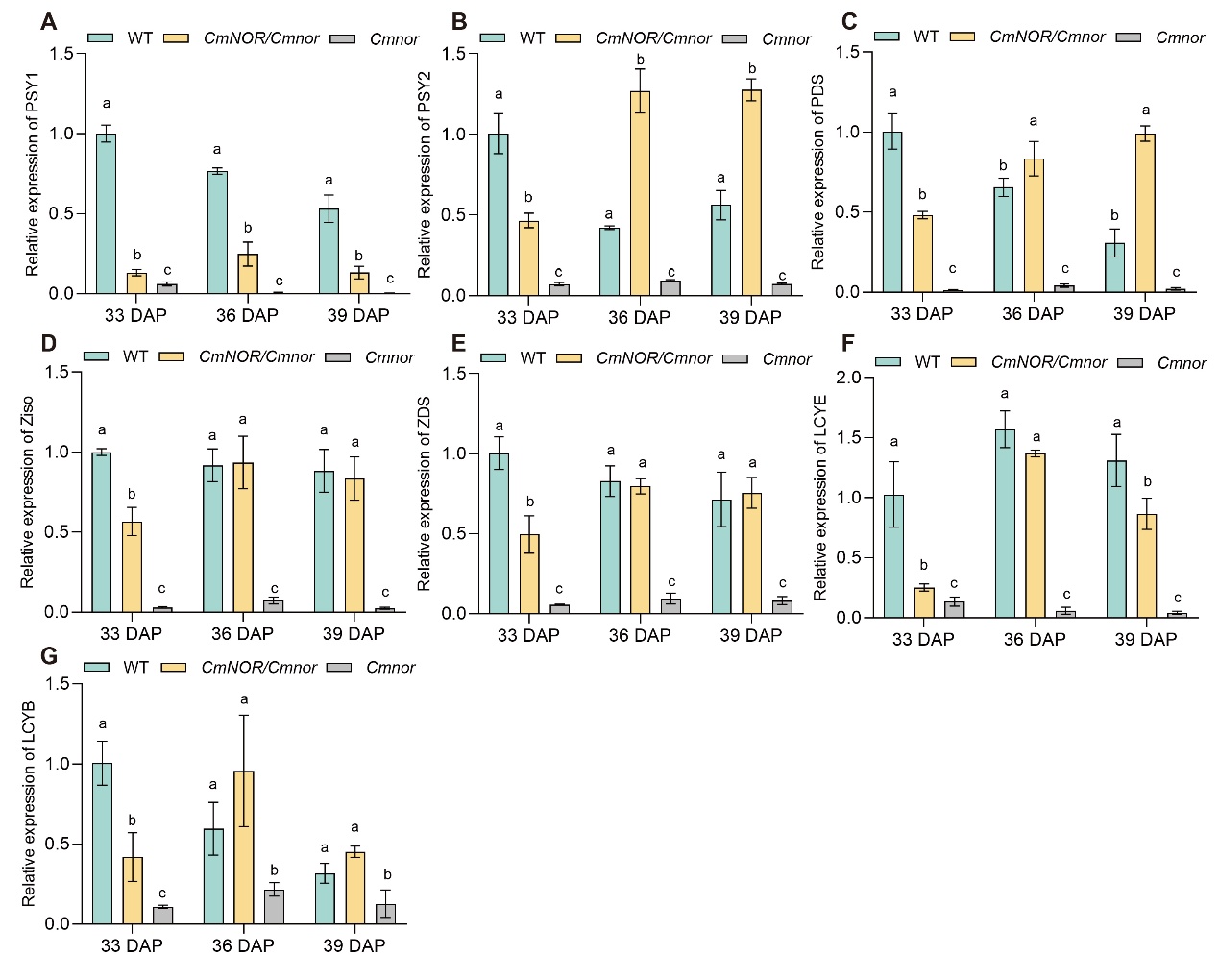


**Figure S4. Relative expression of the key carotenoid biosynthesis genes in the flesh of WT, *CmNOR/Cmnor*, and *Cmnor* fruits at 33, 36, and 39 DAP.**

PSY, phytoene synthase (A, B); PDS, phytoene desaturase (C); ZiSO, 15-cis-zeta-carotene isomerase gene (D); ZDS, zeta-carotene desaturase (E); LCYE, lycopene ε-cyclase (F); LCYB, lycopene β-cyclase (G). Values are means ± SD from three independent replicates. Asterisks denote significant differences compared with WT fruits at each ripening stage. Different lowercase letters indicate significant differences according to Tukey’s multiple range test (p < 0.05).


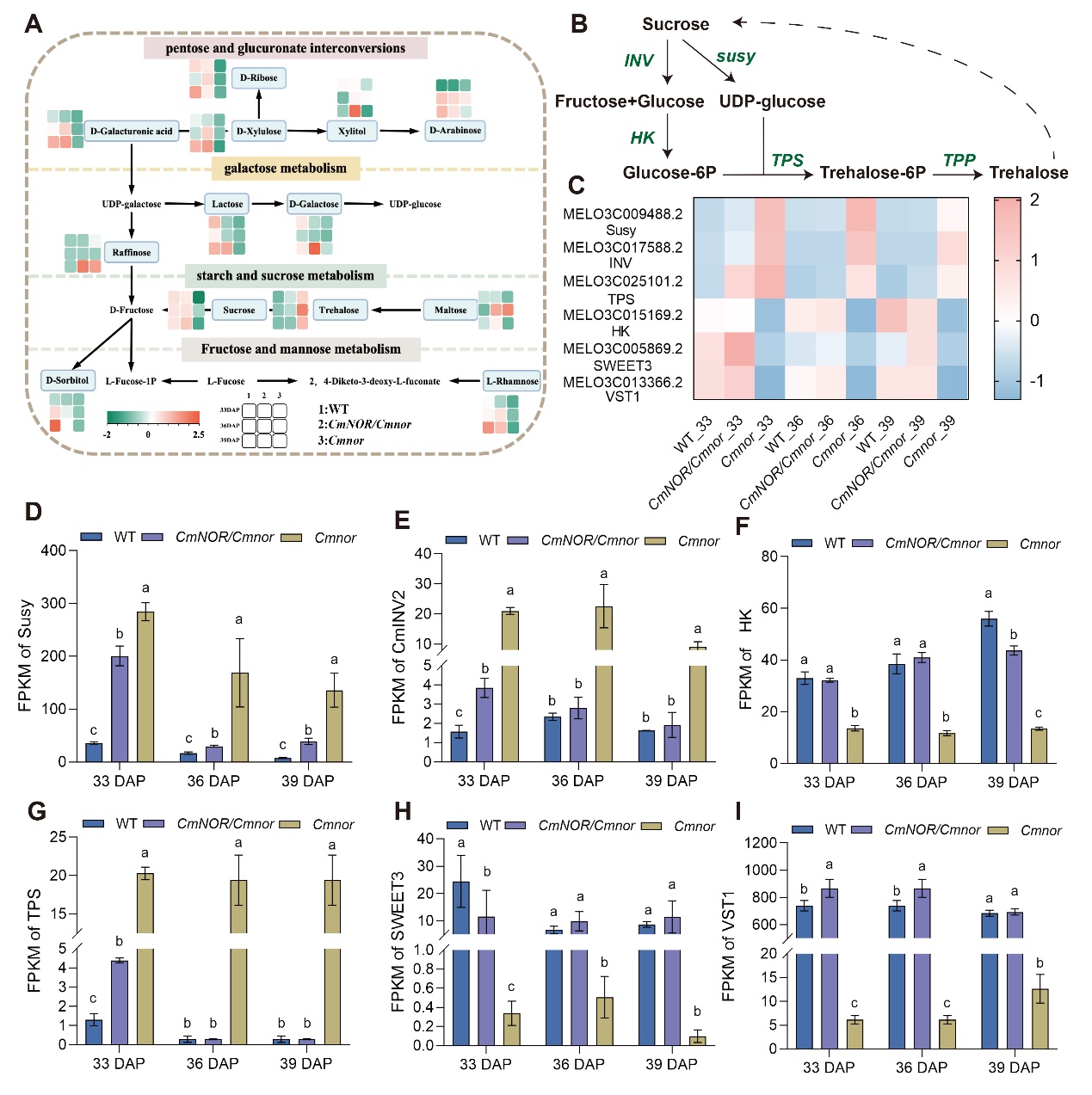


**Figure S5. Analysis of the DEGs in sugar metabolism in the flesh of WT, *CmNOR/Cmnor*, and *Cmnor* fruits at 33, 36, and 39 DAP.**

1. Diagram of the sugar metabolism pathway showing the differentially abundant metabolites in the flesh of WT, *CmNOR/Cmnor*, and *Cmnor* fruits at 33, 36, and 39 DAP. In the heatmap, red indicates higher abundance; blue indicates lower abundance.
2. Diagram of the key genes and metabolites in the sugar metabolism pathway.

**C.** Heatmap representation of expression levels for key sugar metabolism genes in the flesh of WT, *CmNOR/Cmnor*, and *Cmnor* fruits at 33, 36, and 39 DAP.

**D-H.** Expression of key sugar metabolism genes in the flesh of WT, *CmNOR/Cmnor*, and *Cmnor* fruits at 33, 36, and 39 DAP. *Susy*, sucrose synthase (**D**); *CWINV*, cell wall invertase €; *HK*, hexokinase (**F**); *TPS*, trehalose 6-phosphate synthase (**G**); *SWEET*, Sugars Will Eventually Be Exported Transporter (**H**); *VST*, vacuolar sugar transporter (**I**). Values are means ± SD from three independent replicates. Asterisks denote significant differences compared with WT fruits at each ripening stage. Different lowercase letters indicate significant differences according to one-way ANOVA following by Tukey’s multiple range test (*p* < 0.05).

**
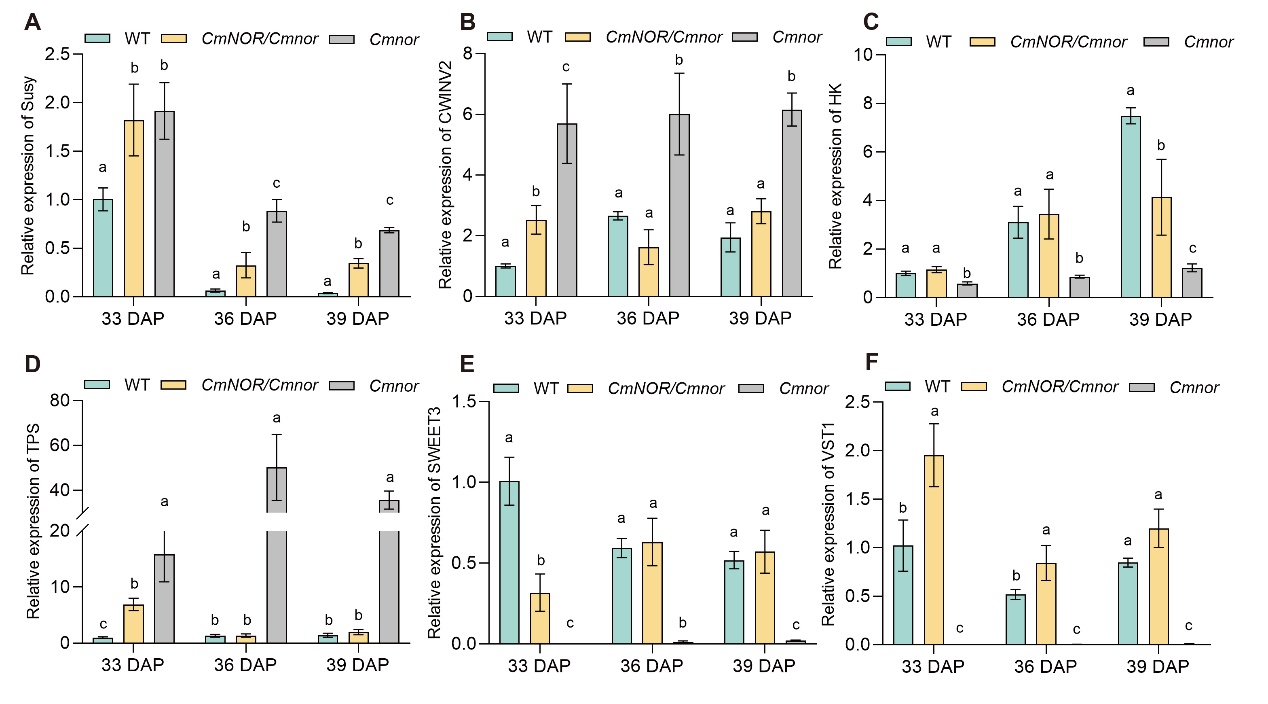
**

**Figure S6.** **Expression of key sugar metabolism genes in the flesh of WT, *CmNOR/Cmnor*, and *Cmnor* fruits at 33, 36, and 39 DAP**.

*Susy*, sucrose synthase (**A**); *CWINV*, cell wall invertase (**B**); *HK*, hexokinase (**C**); *TPS*, trehalose 6-phosphate synthase (**D**); *SWEET*, Sugars Will Eventually Be Exported Transporter (**E**); *VST*, vacuolar sugar transporter (**F**). Values are means ± SD from three independent replicates. Asterisks denote significant differences compared with WT fruits at each ripening stage. Different lowercase letters indicate significant differences according to one-way ANOVA following by Tukey’s multiple range test (*p* < 0.05).


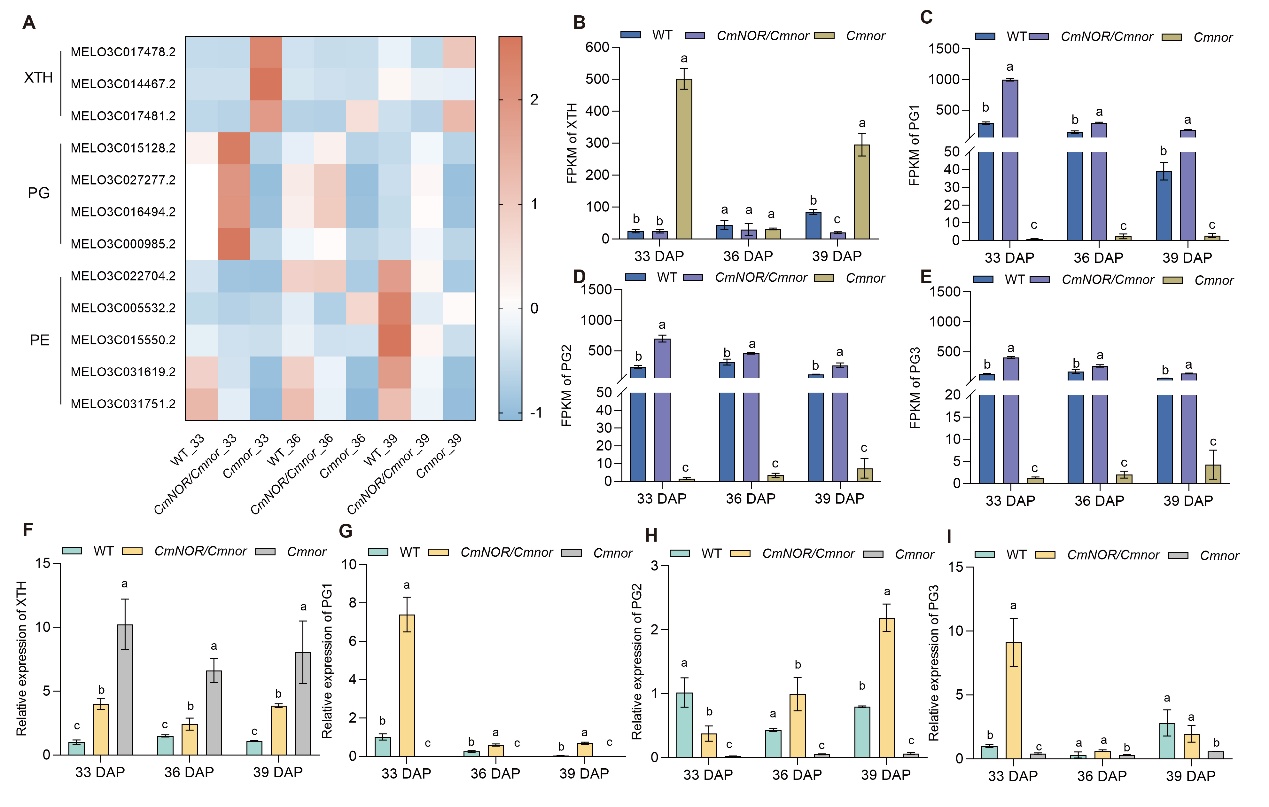


**Figure S7. Expression analysis of DEGs in the cell wall pathway in the flesh of WT, *CmNOR/Cmnor*, and *Cmnor* fruits at 33, 36, and 39 DAP.**

**A**. Heatmap analysis of the key gens in cell wall pathway in the flesh. *XTH*, xyloglucan endotransglucosylase/hydrolase; *PG*, polygalacturonase; *PE*, pectinesterase.

**B-E**. The expression pattern of *XTH* (B) and *PG1-3* (C-E) in the flesh of WT, *CmNOR/Cmnor*, and *Cmnor* fruits at 33, 36, and 39 DAP.

**F-I**. The relative expression pattern of *XTH* (F) and *PG1-3* (G-I) in the flesh of WT, *CmNOR/Cmnor*, and *Cmnor* fruits at 33, 36, and 39 DAP.

Values are means ± SD from three independent replicates. Asterisks denote significant differences compared with WT fruits at each ripening stage. Different lowercase letters indicate significant differences according to Tukey’s multiple range test (p < 0.05).


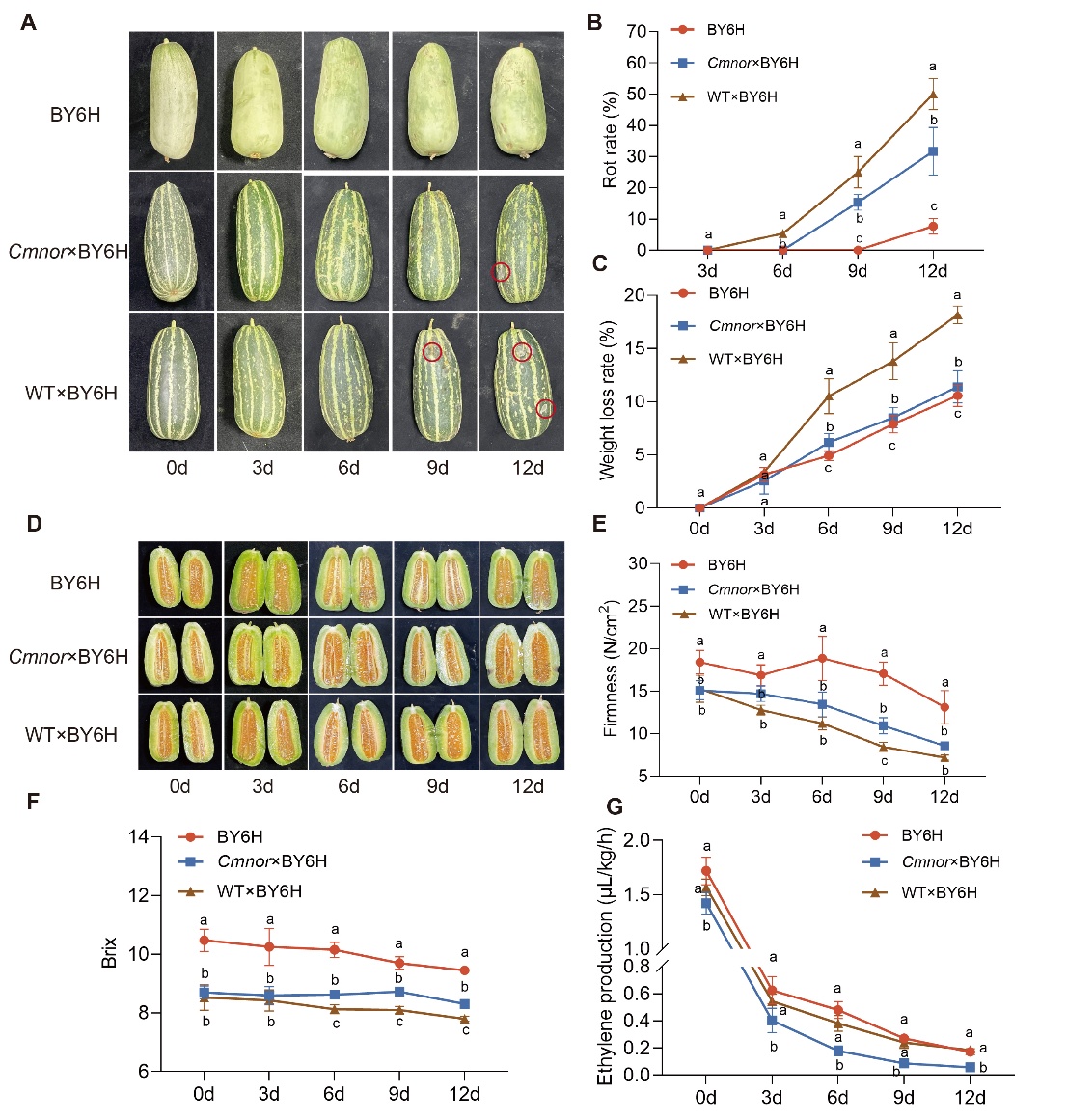


**Figure S8. Phenotypic analysis of fruits from BY6H, *Cmnor* (BY9H)×BY6H F1, and WT×BY6H F1 fruits at 36 DAP.**

1. Representative photographs of fruits from BY6H, *Cmnor* (BY9H)×BY6H F1, and WT×BY6H F1 plants over 12 days of storage.. The red circles indicate rotten sectors.
2. Rot rate of fruits from *Cmnor* (BY9H)×BY6H F1 and WT×BY6H plants over 12 days of storage.
3. Weight loss rate of fruits from *Cmnor* (BY9H)×BY6H F1 and WT×BY6H F1 plants over 12 days of storage.
4. Representative photographs of cut fruits from *Cmnor* (BY9H)×BY6H F1 and WT×BY6H F1 plants over 12 days of storage.
5. Firmness of fruits from *Cmnor* (BY9H)×BY6H F1 and WT×BY6H F1 plants over 12 days of storage.
6. °Brix of fruits from *Cmnor* (BY9H)×BY6H F1 and WT×BY6H F1 plants over 12 days of storage.
7. Ethylene production of fruits from *Cmnor* (BY9H)×BY6H F1 and WT×BY6H F1 plants over 12 days of storage. Values are means ± SD from three replicates. Asterisks denote significant differences compared with WT fruits at each ripening stage. Different lowercase letters indicate significant differences according to one-way ANOVA following by Tukey’s multiple range test (*p* < 0.05).

**
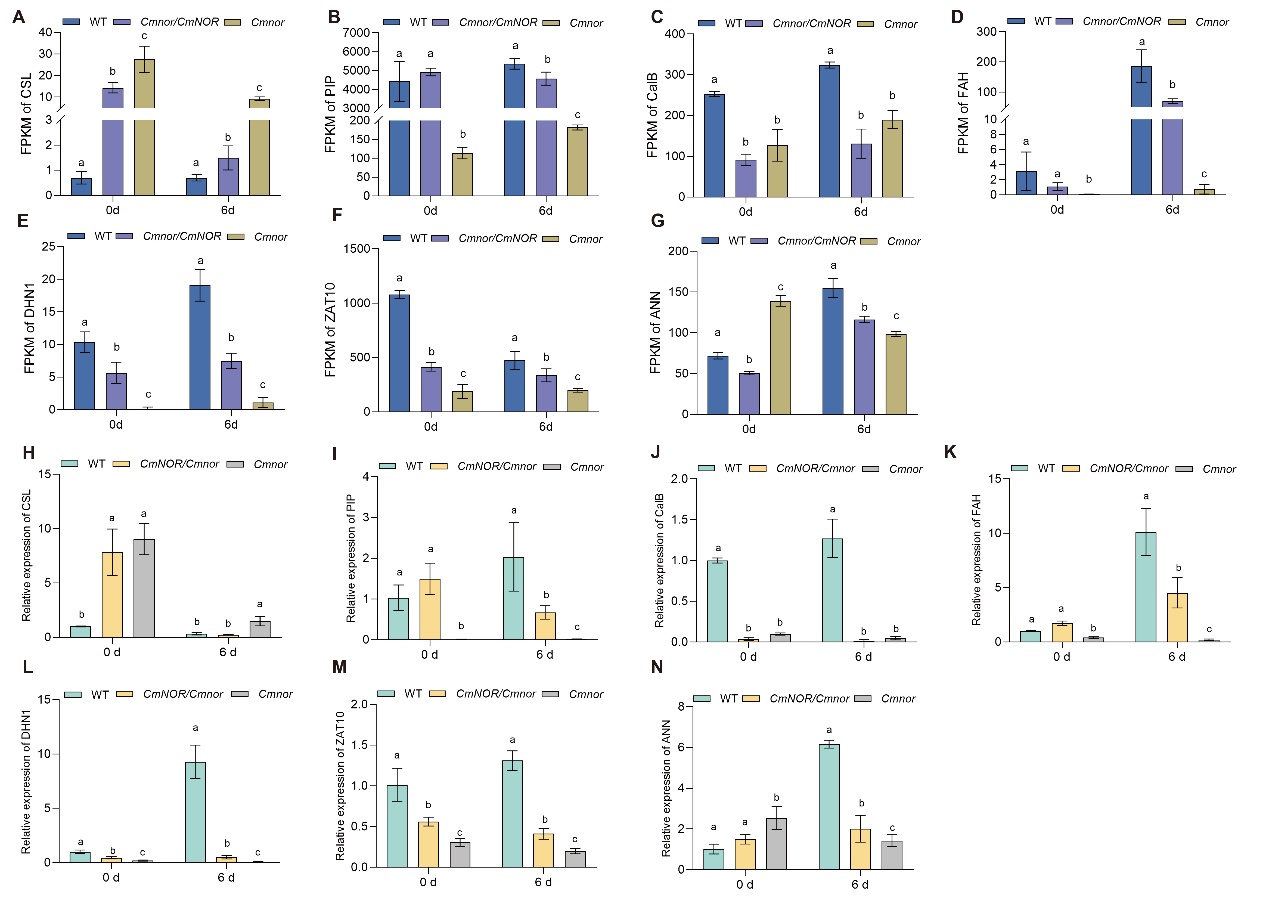
**

**Figure S9. Expression of DEGs related to water deprivation in the flesh of WT, *CmNOR/Cmnor*, and *Cmnor* fruits before and after storage.**

**A-G**. Expression levels of the DEGs related to water deprivation via RNAseq analysis. *CSL*, cellulose synthase like protein (**A**); *PIP*, aquaporin (**B**); *CalB*, calmodulin-binding protein (**C**); *FAH*, fatty acid hydroxylase (**D**); *DHN*, dehydrin (**E**); *ZAT*, zinc finger protein (**F**); *ANN*, annexin (**G**).

**H-N**. Expression levels of the DEGs related to water deprivation via RT-qPCR analysis. *CSL* (**H**); *PIP* (**I**); *CalB* (**J**); *FAH* (**K**); *DHN* (**L**); *ZAT* (**M**); *ANN* (**N**).

Values are means ± SD from three independent replicates. Asterisks denote significant differences compared with WT fruits at each ripening stage. Different lowercase letters indicate significant differences according to one-way ANOVA following by Tukey’s multiple range test (*p* < 0.05).


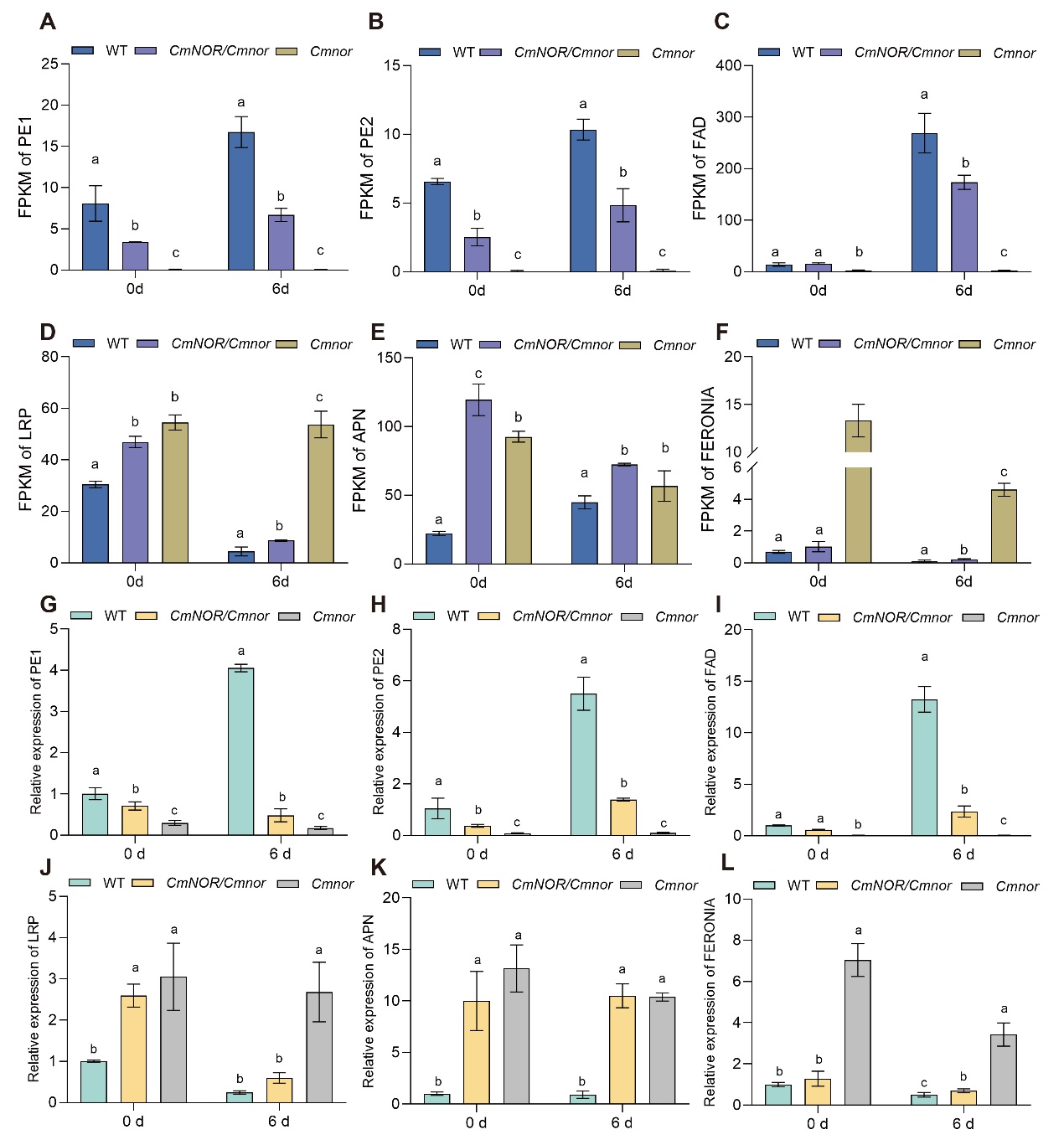


**Figure S10. DEGs related to cell wall constitution in the flesh of WT, *CmNOR/Cmnor*, and *Cmnor* fruits before and after storage.**

1. **F**. Expression levels of the DEGs related to cell wall constitution via RNAseq analysis. *PE1/2*, pectinesterase (**A,B**); *FAD*, FAD-binding Berberine I; *LRP*, leucine-rich repeat receptor (**D**); *APN*, aspartic proteinase nepenthesI(**E**); FERONIA, receptor-like protein kinase (F).

**G-L**. Expression levels of the DEGs related to cell wall constitution via RT-qPCR analysis. *PE1/2* (**G,H**); *FAD* (**I**); *LRP* (**J**); *APN* (**K**); *FERONIA* (**L**).

Values are means ± SD from three independent replicates. Asterisks denote significant differences compared with WT fruits at each ripening stage. Different lowercase letters indicate significant differences according to one-way ANOVA following by Tukey’s multiple range test (*p* < 0.05).


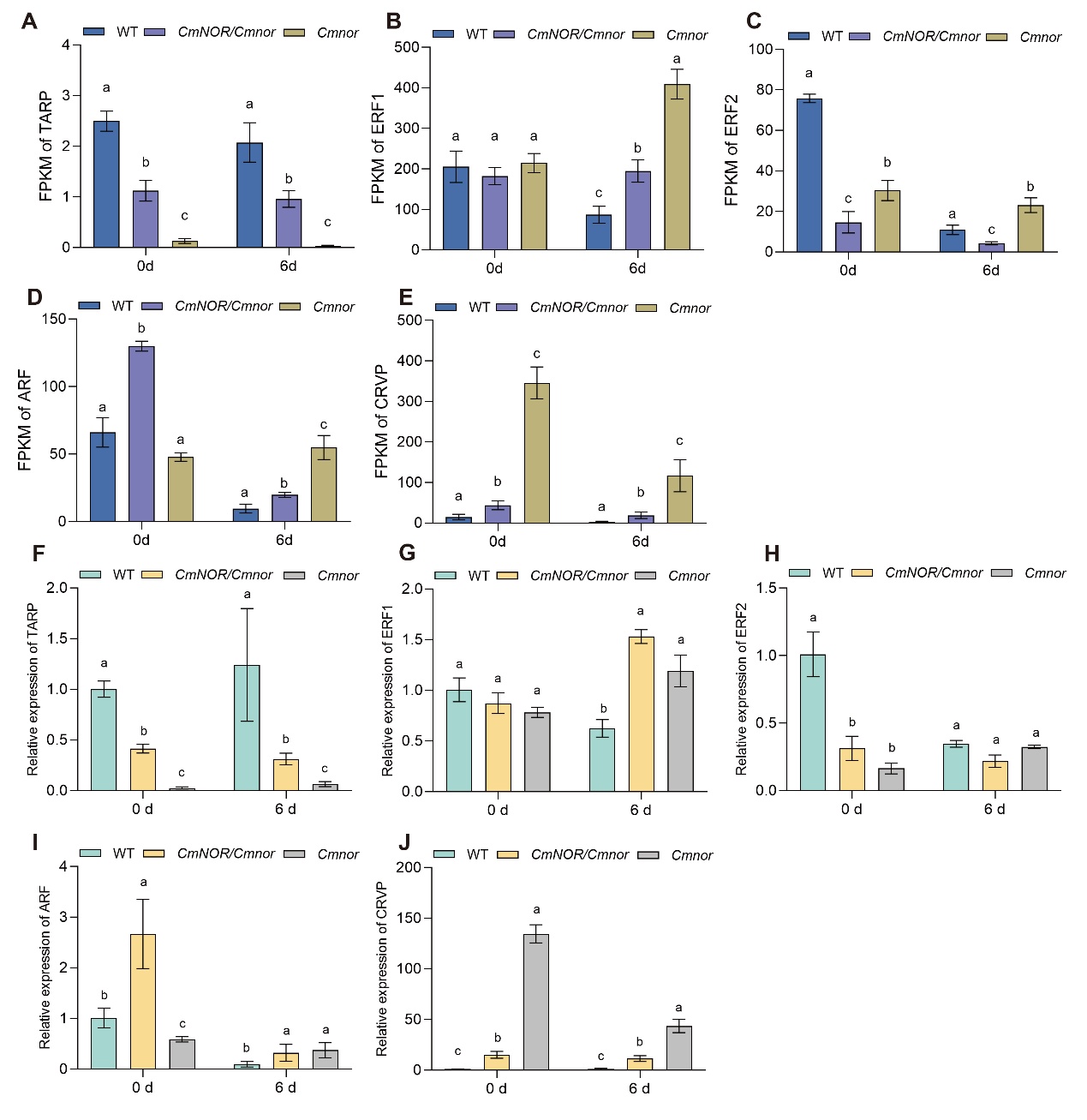


**Figure S11. DEGs related to the ethylene signaling pathway in the flesh of WT, *CmNOR/Cmnor*, and *Cmnor* fruits before and after storage.**

**A-E.** Expression levels of DEGs related to the ethylene signaling pathway via RNAseq analysis. *TATP*, tryptophan aminotransferase-related protein (**A**); *ERF1/2*, ethylene-responsive transcription factors (**B,C**); *ARF*, auxin-responsive family protein (**D**); *CRVP*, cysteine-rich venom protein (**E**).

**F-J.** Expression levels of DEGs related to the ethylene signaling pathway via RT-qPCR analysis. *TATP* (**F**); *ERF1/2* (**G,H**); *ARF* (**I**); *CRVP* (**J**).

Values are means ± SD from three independent replicates. Asterisks denote significant differences compared with WT fruits at each ripening stage. Different lowercase letters indicate significant differences according to one-way ANOVA following by Tukey’s multiple range test (*p* < 0.05).


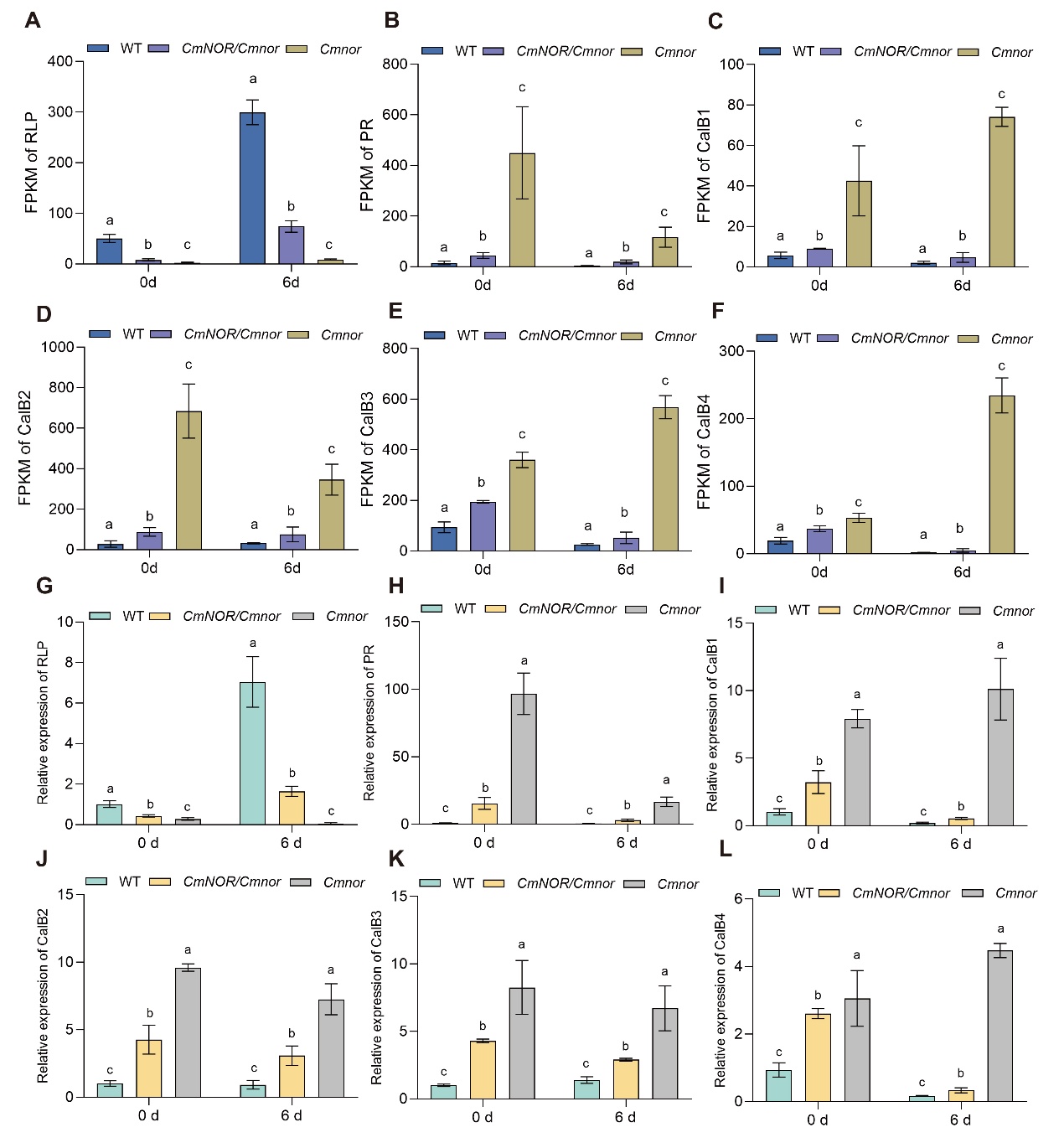


**Figure S12. DEGs related to plant–pathogen interactions in the flesh of WT, *CmNOR/Cmnor*, and *Cmnor* fruits before and after storage.**

**A-F.** Expression levels of DEGs related to plant–pathogen interactions via RNAseq analysis. *RLP*, receptor-like protein kinase (**A**); *PR*, pathogenesis-related protein (B); *CalB1-4*, calcium-binding protein (**C-F**).

**G-L.** Expression levels of DEGs related to plant–pathogen interactions via RT-qPCR analysis. *RLP* (**G**); *PR* (**H**); *CalB1-4* (**I-L**).

Values are means ± SD from three independent replicates. Asterisks denote significant differences compared with WT fruits at each ripening stage. Different lowercase letters indicate significant differences according to one-way ANOVA following by Tukey’s multiple range test (*p* < 0.05).

**Table S1. The primer list used in this study.**

|  | Forward primer: | Reverse primer: |
| --- | --- | --- |
| *CmNOR* | CGCTGAGGTCGATCTCTACAAATTC | CCAGGAGGCTTATTGATACAAGG |
| *CmCYP7* | CGATGTGGAAATTGACGGAA | CGGTGCATAATGCTCGGAA |
| *PSY1* | GATGGACCCCAAATTCCCGATTC | CTCAGCTGAGACGGCTATTTCTC |
| *PSY2* | GATAATTGCAGCACCCAGAAGCAG | CTGCACAGATTTTCCTGCAACTCTC |
| *PDS* | CTGGCAGACATGTTAGTAGGAAACTG | CAGGTTTGTGGCCAGCATCTG |
| *ZiSO* | CGCCGAGGCTTCTATTGGAGAC | CAGCATCCAGAAAGGCCTTCC |
| *ZDS* | GAACCTGAGCGTTATCGAGGAC | CACTTTCCCACCAATGAAGGTCC |
| *LCYE* | GCTTGCTATGGCAAGTGCAGG | CAATAACCACCAAATCCAGTACGCC |
| *LCYB* | GTGCTCTTTTGGAGCTTGTTCCTG | CCCAAACCCCATAATTGTTGGGC |
| *Susy* | AGAGAAAGGGAATAGGAGCTTGAAG | CCAACATCCTGGTTCTGGTCTT |
| *INV* | CAACAGGACCAATGTACTTCAATGG | GGTGTATAAGATGACGGGCTTGTTG |
| *TPS* | GACCTTGATGTTAATGGCGGTTGG | CGGAGAAAGAGTTCCGTCGTAATCC |
| *HK* | GAGTAAGTGGAAGAGAGTGGTCG | CATAAAATGTTCCTTTCTCGCTCCC |
| *SWEET3* | CATTAATGGCCTTGGAATCCTTCTTG | GAAGCAACAAGGCCAATGCATC |
| *VST1* | GTCCAATCCATCTCTGGCAAC | CCAATAAGGCCTTCGATGGAGC |
| *XTH1* | CCAATGGCTTCTTCCTCTGTTTTCTC | GAGTGAGAAGGTCGCCATTGTTG |
| *PG1* | CGTCAATCTTGGAGCCAAGCC | GAAGAAGCCACAAGTGTGCC |
| *PG2* | CTGGCAGACATGTTAGTAGGAAACTG | CAGGTTTGTGGCCAGCATCTG |
| *PG3* | CTCTAATATCGGCACCGGTGACG | CTACATGACTGAACCGTCACATTCTG |
| *CSL* | AGGTGAAGATGACCACTCTAGAGG | CGAGACATGTAAACTAGCATTGGC |
| *PIP* | GACGGACAAGGACTACAAAGAGC | GAATGCCGACGGTGTTGCATTTG |
| *CalB25* | GAAGCTCAATCCCTCACCAGAGC | CAGCTGTAGGGGGAATCGTAG |
| *FAH* | GCGTGATTCACAGTCTCTACCATTAC | AACAGTATTTGATCGTCCCAGTTTCG |
| *DHN1* | GAATACGGCAACGTCATCTCCGAAAC | CATCCTCTGTCGTCTTCGTAATCACC |
| *ZAT* | GCTCTTGAAGCTCTGAATTCTCCTAC | CCTCAGTAGGAGGGTTATCAAATCC |
| *ANN* | CGATATGATGGCCCTGAGGTAAAC | GGTCGGTCTTTAGATCCTTGCTG |
| *PE1* | CTGCACAAAATGTGATTGATTCGC | CTAACATGGATGATCATCCACTGCG |
| *PE2* | GGCAGTACGTATTTGGGCAGAG | GTTAACTGTTTAGCCCATGGTGCAC |
| *FAD* | GGTGCAACGGTTGGTGAAGTTTAC | GTCGACAATCCGAGCATCAATGAC |
| *LRP* | CTGTCATGTCGCCGACCAGAAAG | GTAGAGACTGAGACTAGTGACACG |
| *FERONIA* | GATGAAGGCAATCTCACAGTCGC | CCTCCATGGCAGAAGAGATTTTGAG |
| *APN* | CTAAGAGTTTCATTTACGCACCCTTC | GGATATTTCACCAGCTGCTTCTCC |
| *TARP* | GAATGAGAAGCTCCATAGTGGAGC | CATTGTCGGATCACCATGGTCAAG |
| *ERF1* | CTCTTTGGTCCCACATCGATCCTTTC | CTTCCGAACTTTTCGAGTCTTCTCC |
| *ERF2* | GGACATGGAGGTGCATTTTAGAGG | GTACTACTCCGGCTAGGACTCAG |
| *ARF* | CTCAAGTCGGCCATTAAGAGATG | CGTGACTTACCAACATAGACTGTCC |
| *CRVP* | CCAAGACTCAATCAAAGACTTTGTGG | CTGCCCCATGCAATATTTTCTCC |
| *PR1* | CTCAATCAAAGACTTTGTGGATGCCC | CCATAAGGTCCTTTGGAATGGACTAGG |
| *CalB1* | GACGAAGGAGGAGTTGAAGCAAG | GGCGTACTTAATTAGCTTCCCCATC |
| *CalB2* | CCTGACCAAAGATGAGCTAACTGC | CTTCTAGTGAGATGACCATCACCATC |
| *CalB3* | GCTGTTCTCCTTCAAGGACCG | CACTTCTTCCATTGAACTCCCTCTC |
| *CalB4* | GACTAGGGACGAGGTTAGAGAGATC | GCATGAGAAACACCATAGTGAGC |
| *RLP* | GTAATCCGAGTTCGCCGTTGTC | GGACCGAACCCACCTTCACC |
